# Transcriptome signatures of class I and III stress response deregulation in *Lactobacillus plantarum* reveal pleiotropic adaptation

**DOI:** 10.1186/1475-2859-12-112

**Published:** 2013-11-18

**Authors:** Hermien Van Bokhorst-van de Veen, Roger S Bongers, Michiel Wels, Peter A Bron, Michiel Kleerebezem

**Affiliations:** 1 TI Food & Nutrition, Nieuwe Kanaal 9A, 6709 PA Wageningen, The Netherlands; 2 NIZO food research, P.O. Box 20, 6710 BA Ede, The Netherland; 3 Laboratory of Microbiology, Wageningen University and Research Centre, P.O. Box 8033, 6700 EJ Wageningen, The Netherlands; 4 Centre for Molecular and Biomolecular Informatics, Radboud University Medical Centre, P.O. Box 9101, 6500 HB Nijmegen, The Netherlands; 5 Kluyver Centre for Genomics of Industrial Fermentation, P.O. Box 5057, 2600GA, Delft, The Netherlands; 6 Host-Microbe Interactomics, Wageningen University and Research Centre, P.O. Box 338, 6700 AH Wageningen, The Netherlands; 7 Present address: Food & Biobased Research, Wageningen University and Research Centre, Bornse Weilanden 9, 6708 WG Wageningen, The Netherlands; 8 Present address: Gut Biology and Microbiology, Danone Research, Bosrandweg 20, 6704 PH Wageningen, The Netherlands

**Keywords:** CtsR, HrcA, *Lactobacillus plantarum*, Heat, Stress regulons

## Abstract

**Background:**

To cope with environmental challenges bacteria possess sophisticated defense mechanisms that involve stress-induced adaptive responses. The canonical stress regulators CtsR and HrcA play a central role in the adaptations to a plethora of stresses in a variety of organisms. Here, we determined the CtsR and HrcA regulons of the lactic acid bacterium *Lactobacillus plantarum* WCFS1 grown under reference (28°C) and elevated (40°C) temperatures, using *ctsR*, *hrcA*, and *ctsR*-*hrcA* deletion mutants.

**Results:**

While the maximum specific growth rates of the mutants and the parental strain were similar at both temperatures (0.33 ± 0.02 h^-1^ and 0.34 ± 0.03 h^-1^, respectively), DNA microarray analyses revealed that the CtsR or HrcA deficient strains displayed altered transcription patterns of genes encoding functions involved in transport and binding of sugars and other compounds, primary metabolism, transcription regulation, capsular polysaccharide biosynthesis, as well as fatty acid metabolism. These transcriptional signatures enabled the refinement of the gene repertoire that is directly or indirectly controlled by CtsR and HrcA of *L. plantarum*. Deletion of both regulators, elicited transcriptional changes of a large variety of additional genes in a temperature-dependent manner, including genes encoding functions involved in cell-envelope remodeling. Moreover, phenotypic assays revealed that both transcription regulators contribute to regulation of resistance to hydrogen peroxide stress. The integration of these results allowed the reconstruction of CtsR and HrcA regulatory networks in *L. plantarum*, highlighting the significant intertwinement of class I and III stress regulons.

**Conclusions:**

Taken together, our results enabled the refinement of the CtsR and HrcA regulatory networks in *L. plantarum*, illustrating the complex nature of adaptive stress responses in this bacterium.

## Background

Lactic acid bacteria (LAB) are Gram-positive bacteria that occupy a variety of habitats. LAB are acid tolerant and produce lactate as a major metabolic end-product, thereby generating preservative characteristics to fermented foods and beverages. Due to their long history of use in food products, LAB are generally regarded as safe (GRAS) [1,2]. Next to their prominent role in food fermentation, LAB can be found on plant materials and are among the natural inhabitants of the gastrointestinal (GI) tract of animals and humans [3-5]. Specific *Lactobacillus* strains are marketed as probiotics which are defined as ‘live microorganisms which when administered in adequate amounts confer a health benefit on the host’ [6]. The gastrointestinal tract is the site of action where probiotics are predominantly considered to confer these health benefits, where they may inhibit colonization and infection by pathogens, or may strengthen the intestinal epithelial barrier, or modulate immune responses [7]. Probiotics encounter a variety of stresses during industrial production and storage, e.g. temperature shifts and low water availability during freeze- or spray-drying, or acid stress during storage. Moreover, during GI passage probiotic bacteria are exposed to acid stress in the stomach, as well as exposure to bile salts and digestive enzymes, while they also have to cope with severe nutrient-competition with the endogenous gut microbiota [8].

To persist under stress conditions, probiotics and LAB in general have an arsenal of molecular defense mechanisms [9-12]. Many stress conditions induce protein denaturation and aggregation, and bacteria, including lactobacilli, possess conserved chaperones and proteases to restore or remove misfolded or denatured proteins. This process has extensively been studied in the paradigm Gram-positive bacterium *Bacillus subtilis* using abruptly or constantly elevated temperatures as the inducing stress condition. The repertoire of heat shock responses in *Bacillus subtilis* was stratified in six classes depending on their mode of transcriptional regulation [13-15]. Several of these stress response classes observed in *Bacillus subtilis* are conserved among the LAB, including the highly conserved Class I regulon. Expression of the Class I stress regulon members is controlled by the repressor HrcA, which specifically binds to the inverted repeat element, CIRCE (**c**ontrolling **i**nverted **r**epeat for **c**haperon **e**xpression), under non-stressed conditions. The highly conserved CIRCE element (TTAGCACTC-N9-GAGTGCTAA) is typically found in the promoter regions of the *groE* and *dnaK* operons, which encode the two chaperon complexes GroES-GroEL and HrcA-DnaK-GrpE-DnaJ, respectively [16]. The *hrcA* gene is commonly part of the *dnaK* operon, placing this gene under autorepression control. HrcA-repression is dependent on the availability of the GroELS complex and is relieved when the GroELS chaperon complex is not available, i.e. during stress conditions when non-native proteins arise [13]. The HrcA regulon is not only induced during heat shock, but is also activated by a variety of other stress conditions, including acid, bile, and salt stress [9-11,17]. The genes encompassed within the class III stress regulon appear to be less conserved among LAB, although the class III stress regulon repressor CtsR (**c**lass **t**hree **s**tress gene **r**epressor) appears to be quite consistently present in these bacteria. However, LAB appear to consistently lack the regulatory adaptor genes encoding for McsB and McsA [18,19]. CtsR specifically binds to a heptanucleotide repeat (A/GGTCAAA/T), referred to as the CtsR box [20]. This *cis*-acting regulatory element is commonly encountered in the promoter regions of *clpP* and several other, but not all, *clp* genes, which encode Clp-proteases that are involved in protein quality control during both stress and non-stress conditions [21]. ClpP mediated proteolysis removes misfolded proteins from the cell, but Clp proteases can also function in cellular differentiation processes [21]. In some organisms other transcription regulators, including HrcA, are involved in co-regulation of the CtsR target genes [21,22]. In conclusion, HrcA and CtsR are key components in stress response regulation, which may include cross-regulation between their respective regulons.

*Lactobacillus plantarum* is encountered in several environmental niches, including fermented foods and the human GI tract, and specific strains are marketed as probiotics [23]. *L. plantarum* WCFS1, a single colony isolate of strain NCIMB 8826, has been shown to actively survive passage through the human digestive tract [24,25], and it was the first *Lactobacillus* species of which the complete genome sequence was determined [26]. Besides the genome sequence, advanced functional annotations, as well as sophisticated bioinformatics and mutagenesis tools have been developed, enabling the investigation of gene-regulatory mechanisms at the molecular level [27-29]. For example, the *hrcA* and *ctsR* regulon members could be predicted on basis of the conserved *cis*-acting elements involved, which has in part been confirmed experimentally [11,30-33]. Some of the HrcA and CtsR regulon members in *L. plantarum* WCFS1 have been detected through phylogenetic footprinting [32], large scale analysis of co-regulation of expression [33], or via DNA binding assays [30,31]. Moreover, gene-expression responses in *L. plantarum* have been unraveled for various stress conditions, including lactate [34], low pH [34], oxidative [35,36], solvent [37,38], bile [39], cold [37], and heat stress [37]. Analysis of available transcriptome data indicates that some but not all of the predicted HrcA and CtsR regulon members of *L. plantarum* WCFS1 are differentially expressed during these different stress challenges [33]. Despite the characterization of these stress responses, the exact regulons of HrcA and CtsR in *L. plantarum* remain not completely determined.

This paper describes the regulons of CtsR and HrcA at reference and elevated growth temperatures by determination of the whole-genome transcriptome patterns of *ctsR*, *hrcA*, and *ctsR*-*hrcA* deletion mutants [38]. The data revealed that the CtsR or HrcA deficient strains displayed altered transcription patterns of genes encoding functions involved in transport and binding of sugars and other compounds, primary metabolism, as well as cell envelope remodeling. Moreover, deficiency of both transcription factors elicited temperature-dependent and pleiotropic transcriptional adaptation of the cell. Finally, stress-phenotyping of the mutants revealed a role of both regulators in the regulation of oxidative stress tolerance.

## Materials and methods

### Strains and growth conditions

*L. plantarum* WCFS1 [26], Δ*ctsR* (NZ3410) [38], Δ*hrcA*::*cat* (NZ3425^CM^) [38], and Δ*ctsR*Δ*hrcA*::*cat* (NZ3423^CM^) [40] were grown in MRS (de Man-Rogosa-Sharpe) broth (Difco, West Molesey, United Kingdom) in pH-controlled batch fermentations at 0.5 L scale in a Multifors mini-in parallel fermentor system (Infors-HT Benelux, Doetinchem, the Netherlands). A single colony isolate of *L. plantarum* WCFS1 or its derivatives was used to inoculate 5 mL of MRS followed by overnight growth at 37°C. The full-grown culture was used to prepare a dilution range from 10^-1^ to 10^-6^ in fresh medium and these dilutions were grown overnight. Subsequently, the culture density was assessed by determination of the optical density at 600 nm (OD_600_) and the culture that had an OD_600_ closest to 1.5 (representing logarithmically growing cells) was used to inoculate the fermentors at an initial OD_600_ of 0.1. During fermentation the cultures were stirred at 125 rpm, the pH of the culture was maintained at 5.8 by titration of 2.5 M NaOH, and temperature was set at 28°C or 40°C. A biologically independent duplicate; i.e., derived from independent colonies and performed on separate days, was included for all strains and temperatures. Cells were harvested at an OD_600_ of 1.0 for RNA isolation.

### RNA isolation and microarray analysis

RNA extraction, labeling and hybridization, as well as data analysis were performed as described previously [41,42]. Briefly, following quenching and cell disruption by bead beating, RNA was isolated using the High Pure kit including 1 h treatment with DNaseI (Roche Diagnostics, Mannheim, Germany). The resulting RNA was reverse transcribed to obtain cDNAs which were labeled using Cyanine 3 or Cyanine 5 labels (AmershamTM, CyTMDye Post-labelling Reactive Dye Pack, GE Healthcare, UK). The cDNAs were hybridized (Additional file 1: Figure S1) on WCFS1-specific, custom-made Agilent arrays. Each microarray contained at least 2, but mostly 3 distinct probes for all of the genes detected within the genome. These probes were spotted in duplicate on each array, which was based on the Agilent 15 k format (GEO accession number GPL13984; http://www.ncbi.nlm.nih.gov/geo/). Subsequently, the slides were washed and scanned using routine procedures [41,42] and the obtained transcriptome profiles were normalized using Lowess normalization [43]. The data were corrected for inter-slide differences on the basis of total signal intensity per slide using Postprep [44]. The median intensity of the different probes per gene was selected as the gene expression intensity. This analysis resulted in genome-wide, gene expression levels for *L. plantarum* WCFS1, NZ3410, NZ3423^CM^, and NZ3425^CM^. CyberT was used to compare the different transcriptomes [45]. This analysis resulted in a gene expression ratio and false discovery rate (FDR) for each gene. Genes were considered significantly differentially expressed when FDR-adjusted p-values were < 0.05. The DNA microarray data is available under GEO accession number GSE31253.

### Data analysis tools

Visualization of the genes displaying differential expression in the mutants as compared to the wild-type was performed by loading Excel files into the Cytoscape software suite [46]. Data were first ordered using the spring embedded sorting algorithm in the Cytoscape tool. Coloring of the edges (up- or downregulation of the mutants over wild type) and nodes (annotated main class) and structuring of the network were performed manually. The SimPheny™ software package (Genomatica InC., San Diego, USA) loaded with the *L. plantarum* WCFS1 genome-scale model [28] was used to visualize differentially expressed genes that encode enzymes in metabolic pathways. Over-represented main classes and subclasses in the transcriptome data were identified using the Biological Networks Gene Ontology (BiNGO) [47] Cytoscape plugin. MEME software [48] was used with default settings to predict conserved *cis*-acting motifs from 300 nt upstream regions preceding the predicted translation start of the first genes of the operons of all genes. Subsequently, MAST [49] was used to perform genome-wide searches for the MEME-predicted *cis*-acting elements of HrcA and CtsR [32,33].

### Phenotypic assays

To determine growth efficiency of the different mutant strains, *L. plantarum* WCFS1 or its derivatives were grown in MRS at 28°C, 37°C, 40°C, or 42°C, and growth was monitored by OD_600_ measurement during 72 hours (SPECTRAmax PLUS384, Molecular Devices, UK). The maximum specific growth rate was determined by taking the slope of 5 consecutive ln transferred OD data points that gave the highest number. To quantify the colony forming capacity at elevated temperature, the wild type and gene deletion derivatives were grown at 30°C, serially diluted on MRS agar plates, and incubated for 1 week at 30°C or 42°C. Hydrogen peroxide stress tolerance was measured as described before [38]. In short, PBS washed cultures (OD_600_ = 1.0) were resuspended in PBS containing 40 mM hydrogen peroxide at RT and samples were taken from this suspension, every 5 min for 60 min, and colony forming units were enumerated by plating of serial dilutions. Bile resistance was monitored as described before [50]. Briefly, cultures were inoculated in MRS containing 0.1% porcine bile (Sigma, Zwijndrecht, The Netherlands) at 28°C and growth was monitored by OD_600_ determination (SPECTRAmax PLUS384, Molecular Devices, UK). H_2_O_2_ inactivation data were compared by fitting a reparameterized Weibull model according to Metselaar *et al*. [51]:

$$\mathrm{lo}g_{10}\left(N_{t}\right)=\mathrm{lo}g_{10}\left(N_{0}\right)-\Delta {\left(\frac{t}{t_{\mathrm{\Delta D}}}\right)}^{\beta }$$

in which *Δ* is the number of decimal reductions, *t*_*ΔD*_ the time needed to reduce the initial number of microorganisms with *Δ* decimals (min), and *β* a fitting parameter that defines the shape of the curve. *Δ* was set at 4 and the other parameters were estimated using Excel 2010. Two-sided Student’s *t*-test was used for statistical analysis and *p* < 0.05 was considered significant.

## Results

### HrcA and CtsR are involved in the heat stress response of *L. plantarum*

**Figure 1:**
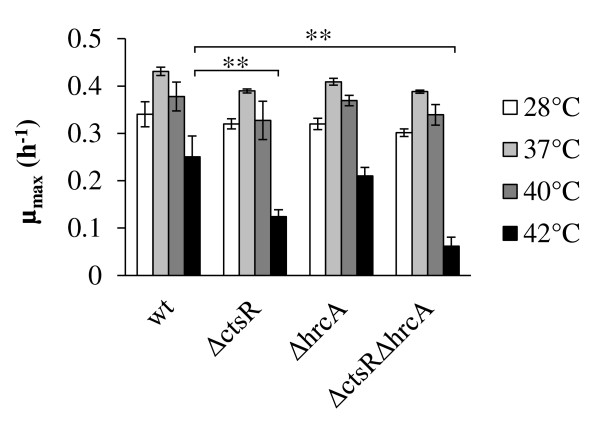
**Maximum specific growth rates of *L*. *plantarum *WCFS1 (wt), NZ3410 (Δ*ctsR*), NZ3425**^**CM **^**(Δ*hrcA*), and NZ3423**^**CM **^**(Δ*ctsR*Δ*hrcA*).** Specific growth rates are shown for reference (28°C) and elevated (37°C, 40°C, and 42°C) temperatures as indicated in the figure legend. Asterisks indicate *p*-value < 0.001. Data shown are mean ± standard deviation of 3 independent experiments.

**Figure 2:**
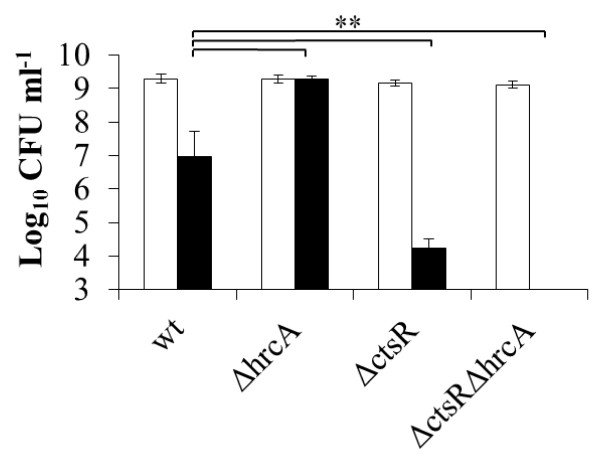
**Involvement of CtsR and HrcA in the ability to form colonies at elevated temperature.***L. plantarum* WCFS1 (wt), NZ3410 (Δ*ctsR*), NZ3425^CM^ (Δ*hrcA*), and NZ3423^CM^ (Δ*ctsR*Δ*hrcA*) cultures were serial diluted on MRS plates and incubated at control (30°C; white bars) or elevated temperature (42°C; black bars). Asterisks indicate *p*-value < 0.001. Data shown are mean ± standard deviation of 3 independent experiments.

HrcA and CtsR are regulators of class I and class III stress responses, respectively, including heat induced stress [13]. The role of these repressors at reference and elevated temperature was investigated in *L. plantarum* and its previously constructed derivatives that are deficient in either CtsR or HrcA alone, or both [38]. The maximum specific growth rate of the Δ*ctsR*, Δ*hrcA*::*cat*, and Δ*ctsR*Δ*hrcA*::*cat* strains at 28, 37, and 40°C did not differ from the *L. plantarum* WCFS1 wild-type strain (Figure 1). These findings expand earlier observations demonstrating unaltered growth characteristics of another *L. plantarum ctsR* mutant relative to its parental strain at 28°C [31]. However, although the maximum specific growth rate of Δ*hrcA*::*cat* was comparable to the wild-type at 42°C, the Δ*ctsR* and Δ*ctsR*Δ*hrcA*::*cat* mutants displayed 2.0- and 4.1-fold (*p* < 0.001; Figure 1) decreased specific growth rates, respectively. This result indicates that CtsR is required to sustain normal specific growth rates at 42°C. When serial dilutions of stationary phase cultures grown at 30°C were spotted on MRS plates, followed by continued incubation at 30°C, the wild-type and mutant strains gave approximately equal numbers of colonies, which were in all cases within the range anticipated for full-grown cultures. This observation indicates that HrcA and CtsR do not influence the colony forming unit (CFU) numbers of *L. plantarum* WCFS1 at 30°C. Notably, when the plates were incubated at 42°C, the wild type strain generated approximately 100-fold lower CFU as compared to incubation at 30°C (*p* < 0.001). Importantly, the CFU numbers obtained with the Δ*ctsR* mutant were even stronger reduced at 42°C (*p* < 0.001), and this effect was even more pronounced for the Δ*ctsR*Δ*hrcA*::*cat* mutant (Figure 2). Conversely, CFU numbers for the mutant lacking a functional *hrcA* were not significantly different at 30°C, and 42°C, indicating that this mutation contributes to increased robustness as compared to the wild-type at this elevated temperature (Figure 2).

### Transcriptional response of L. *plantarum* during heat stress

To investigate the transcriptional response of *L. plantarum* to elevated temperature and the role of CtsR and HrcA herein, transcriptome profiles of *L. plantarum* WCFS1 at control and elevated temperatures were determined. The control temperature of 28°C and elevated temperature of 40°C were selected since *L. plantarum* wild type displays similar specific growth rates at these temperatures as compared to the CtsR and HrcA deficient derivatives (see above). This prevents blurring of the results by genes responding to differential specific growth rates. When comparing the transcriptomes obtained for the wild-type strain at the two temperatures, more than 1000 genes were significantly differentially expressed and 488 genes (exclusive genes with phage and prophage related functions) were more than 2-fold upregulated or downregulated (Additional file 2: Table S1). At 40°C *hrcA* expression was reduced, while that of *groEL* and *groES* were induced. In addition, *clpP*, *clpB*, and *clpE*, expression levels were induced at the elevated temperature. Of the other (predicted) HrcA or CtsR regulon members (see Table 1) only *hsp1* (small heat shock protein 1, which has been shown to be regulated by CtsR [31] and is also predicted to be regulated by HrcA [11]) was induced. In addition, at 40°C many genes coding for proteins with regulatory functions were transcribed at an elevated level, suggesting that their regulons contribute to maintenance of normal specific growth rates at this elevated growth temperature, while genes coding for proteins involved in degradation of proteins, peptides, and glycopeptides were repressed. Other transcriptional changes observed at elevated temperature were the downregulation of the capsular polysaccharide (*cps*)-clusters *1*, *3*, and *4*, while many cell surface proteins, including *cscII*, encoding one of 9 cell surface complexes (*lp*_*2173*-*lp*_*2175* (50)) were upregulated. Moreover, the majority of genes required for membrane lipid biosynthesis were down-regulated, including genes encoding fatty acid elongation proteins (*fab*), acyl carrier proteins (ACP), and acetyl-CoA carboxylases (ACC). The *fab*-locus encompasses 13 genes, which were all repressed at least 3.3-fold. In addition, expression levels of *dak1A*, involved in glycerolipid metabolism, and cyclopropane-fatty-acyl-phospholipid synthase (*cfa*-*1*) were increased, while its paralogue *cfa*-*2* was repressed. These results strongly suggest that *L. plantarum* adapts its cell envelope in response to growth at elevated temperature.

**Table 1:** **Fold-changes of predicted and verified CtsR and HrcA regulon members**^**a **^**in the NZ3410 (Δ *ctsR *), NZ3425**^**CM **^**(Δ *hrcA *:: *cat *), and NZ3423**^**CM **^**(Δ *ctsR *** **Δ *hrcA *:: *cat *) strains compared with the wild type**

**ID**^**b**^	**Name**	**Function**	***p*-value**^**c**^	**28°C**			**40°C**		
				**Δ** ***ctsR***	**Δ** ***hrcA***	**Δ** ***ctsR *** **Δ** ***hrcA***	**Δ** ***ctsR***	**Δ** ***hrcA***	**Δ** ***ctsR *** **Δ** ***hrcA***
**CtsR**									
lp_0786	*clpP*	Endopeptidase Clp, proteolytic subunit		**2.42**^**d**^	−1.09	**2.33**	1.27	−1.29	1.12
lp_1269	*clpE*	ATP-dependent Clp protease, ATP-binding subunit ClpE	2.0·10^-10^	**2.27**	−1.02	**2.12**	−1.01	−1.20	−1.12
lp_1903	*clpB*	ATP-dependent Clp protease, ATP-binding subunit ClpB	5.0·10^-10^	**7.01**	1.00	**6.92**	**4.24**	−1.33	**3.71**
lp_1018	*ctsR*	transcription repressor of class III stress genes		**−696**	−1.15	**−984**	**−526**	−1.31	**−161**
lp_1019	*clpC*	ATP-dependent Clp protease, ATP-binding subunit ClpC		**1.92**	−1.09	**1.84**	**1.76**	−1.30	**1.58**
lp_0129	*hsp1*	Small heat shock protein	3.9·10^-11^	**5.57**	**3.16**	**12.70**	1.12	**−1.38**	1.21
lp_2945	*lp*_*2945*	Aromatic acid carboxylyase, subunit C (putative)	3.5·10^-10^	1.27	−1.08	1.57	1.21	1.20	1.46
lp_2451	*lp*_*2451*	Prophage P2a protein 6; endonuclease	4.9·10^-7^	1.05	1.11	1.12	1.03	**1.40**	1.32
lp_2926	*lp*_*2926*	Unknown	2.8·10^-6^	1.08	−1.08	−1.10	1.30	−1.19	1.05
lp_2426^e^	*lp*_*2426*	Prophage P2a protein 31; phage transcriptional regulator, ArpU family	2.8·10^-6^	−1.18	−1.56	−2.07	**8.85**	−1.87	1.31
lp_2540	*lp*_*2540*	Unknown	4.0·10^-6^	1.09	−1.31	4.11	−1.27	1.27	−1.14
lp_2541	*lp*_*2541*	ABC transporter, substrate binding protein	4.0·10^-6^	−1.15	−1.03	1.01	1.07	1.31	**1.44**
lp_2542	*lp*_*2542*	ABC transporter, permease protein (putative)	4.0·10^-6^	−1.03	−1.12	−1.06	−1.02	1.09	1.15
lp_2543	*lp*_*2543*	ABC transporter, ATP-binding protein	4.0·10^-6^	−1.18	1.02	**1.27**	−1.14	1.15	1.01
lp_3530	*treP*	Trehalose phosphorylase	4.0·10^-6^	−1.20	−1.25	−1.05	2.30	−1.32	−1.13
lp_2061	*lp*_*2061*	Unknown	4.0·10^-6^	**1.38**	**1.53**	**1.47**	−1.21	1.10	1.07
lp_2029	*hrcA*	Heat-inducible transcription repressor HrcA	5.8·10^-6^	−1.32	**−241**	**−147**	1.15	**−176**	**−145**
lp_2028	*grpE*	Heat shock protein GrpE	5.8·10^-6^	−1.04	1.48	1.23	−1.21	1.26	−1.27
lp_2027	*dnaK*	Chaperone, heat shock protein DnaK	5.8·10^-6^	−1.23	1.30	1.16	−1.28	1.09	**−1.43**
lp_2842	*lp*_*2842*	Transcription regulator, LysR family	6.7·10^-6^	1.08	1.14	−1.04	−1.17	−1.34	1.03
lp_1843	*lp*_*1843*	Aldose 1-epimerase family protein	9.8·10^-6^	−1.06	−1.14	1.06	**1.50**	1.19	1.20
lp_1845	*hslU*	ATP-dependent Hsl protease, ATP-binding subunit HslU	9.8·10^-6^	1.10	−1.02	1.23	**1.65**	1.08	**1.44**
lp_1846	*hslV*	ATP-dependent protease HslV	9.8·10^-6^	1.16	1.14	**1.31**	**1.78**	1.11	**1.50**
lp_1847	*lp*_*1847*	Integrase/recombinase, XerC/CodV family	9.8·10^-6^	1.22	1.22	**1.36**	**1.73**	1.11	**1.36**
**HrcA**									
lp_0727	*groEL*	GroEL chaperonin	5.9·10^-9^	−1.19	**2.00**	**1.59**	−1.46	1.06	−1.50
lp_0728	*groES*	GroES co-chaperonin	5.9·10^-9^	−1.21	**2.13**	**1.62**	−1.55	1.14	−1.50
lp_2029	*hrcA*^*f*^	Heat-inducible transcription repressor HrcA	2.9·10^-14^	−1.32	**−241**	**−147**	1.15	**−176**	**−145**
lp_2028	*grpE*	Heat shock protein GrpE	2.9·10^-14^	−1.04	1.48	1.23	−1.21	1.26	−1.27
lp_2027	*dnaK*	Chaperone, heat shock protein DnaK	2.9·10^-14^	−1.23	1.30	1.16	−1.28	1.09	**−1.43**
lp_2026	*dnaJ*	Chaperone protein DnaJ		−1.13	1.05	1.17	−1.07	1.08	1.14
lp_0726	*lp*_*0726*	Membrane-bound protease, CAAX family	1.0·10^-7^	**1.90**	−1.07	**1.56**	**2.26**	−1.22	**2.44**
lp_0129	*hsp1*	Small heat shock protein		**5.57**	**3.16**	**12.70**	1.12	**−1.38**	1.21
lp_0413	*plnQ*	Plantaricin biosynthesis protein PlnQ	6.9·10^-7^	−1.03	1.23	1.51	−2.14	−1.16	1.16
lp_3578	*kat*	Catalase	1.0·10^-6^	1.02	1.02	1.03	1.28	−1.20	−1.13
lp_3617	*tal3*	Transaldolase	1.7·10^-6^	−1.19	−1.04	1.22	1.26	1.14	−1.28
lp_3618	*pts37A*	Sorbitol PTS, EIIA	1.7·10^-6^	1.03	1.02	1.33	**4.51**	1.33	1.26
lp_3619	*pts37BC*	Sorbitol PTS, EIIBC	1.7·10^-6^	2.15	1.31	2.50	2.68	−1.40	−1.23
lp_3620	*pts37C*	Sorbitol PTS, EIIC	1.7·10^-6^	1.00	−1.33	−1.10	1.88	1.19	1.46
lp_3621	*srlM1*	Sorbitol operon activator	1.7·10^-6^	1.39	1.17	2.13	2.22	1.08	1.40
lp_3622	*srlR1*	Sorbitol operon transcription antiterminator, BglG family	1.7·10^-6^	−1.36	−1.13	−1.05	2.17	1.01	1.30
lp_3623	*srlD1*	Sorbitol-6-phosphate 2-dehydrogenase (EC 1.1.1.140)	1.7·10^-6^	−1.10	−1.35	−1.43	1.37	−1.16	1.88
lp_1268	*lp*_*1268*	Integrase/recombinase	3.7·10^-6^	**−2.21**	−1.10	**−1.56**	**−3.07**	1.49	**−3.38**
lp_0387	*lp*_*0387*	Unknown	2.4·10^-6^	1.18	1.04	1.25	1.06	−1.00	1.35
lp_1879	*hbsU*	DNA-binding protein	9.9·10^-6^	−1.14	1.04	−1.14	−1.23	1.04	**−1.27**
lp_1880	*lp*_*1880*	Unknown	9.9·10^-6^	−1.13	1.11	−1.14	**−1.59**	1.20	**−1.71**

^a^Adapted from [1].

^b^The lp_number indicates gene number on *L. plantarum* WCFS1 chromosome [2].

^c^*p*-value of the best match on the upstream sequence after comparing the canonical regulatory factor binding site. Values lower than 1.0·10^-5^ were included.

^d^Fold-changes in bold are significant (FDR adjusted *p*-value < 0.05).

^e^The *cis*-element is predicted to be in front of this operon that contains *lp*_*2426* until *lp*_*2431*, which all encode proteins of prophage P2a. Fold-changes are only given for *lp*_*2426*.

^f^The upstream region of *hrcA* contains two CIRCE elements. The second has a *p*-value of 8.3·10^-9^.

### Impact of CtsR and HrcA deficiency on expression of their predicted regulons members

**Figure 3:**
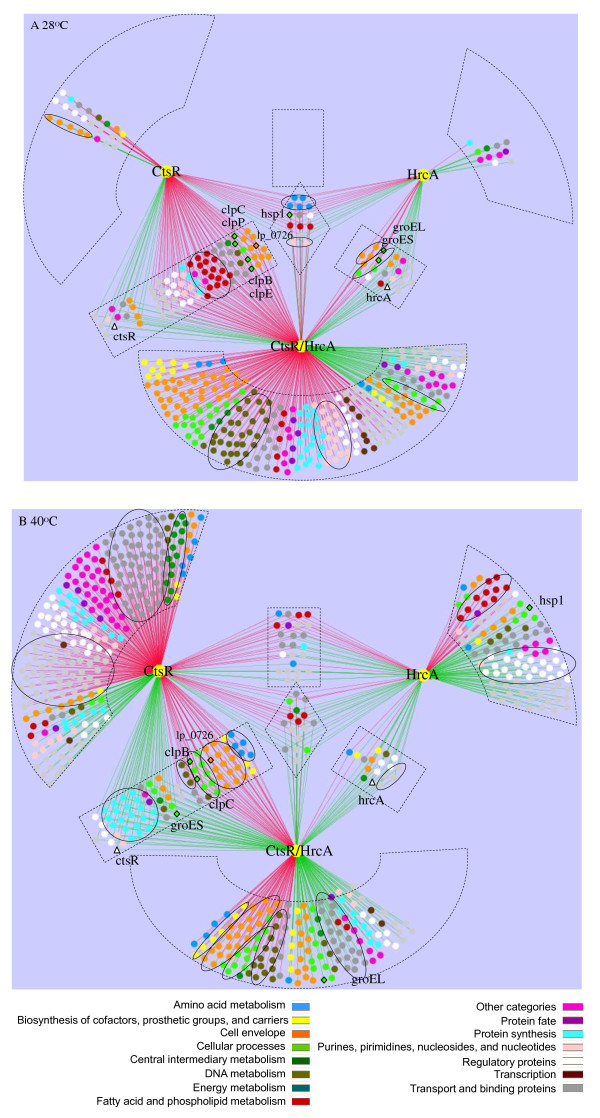
**Significantly differentially transcribed genes in NZ3410 (Δ*ctsR*), NZ3425**^**CM **^**(Δ*hrcA*), and NZ3423**^**CM **^**(Δ*ctsR*Δ*hrcA*) as compared to the wild-type grown at 28°C (A) or 40°C (B).** Yellow colored octangular nodes represent the mutants and other colored nodes indicate main classes. The red and green lines indicate up- or downregulation, respectively. Triangle nodes indicate the CtsR or HrcA transcription regulator, diamond nodes indicate genes that are predicted to be part of the CtsR and/or HrcA regulon, whereas black ovals indicate over-represented main classes or subclasses in that particular main class. The main class “hypothetical proteins” was excluded.

To unravel the role of HrcA and CtsR regulation in adaptation to growth at elevated temperatures, we evaluated the transcriptome profiles of the Δ*ctsR*, Δ*hrcA*::*cat*, and Δ*ctsR*Δ*hrcA*::*cat* mutants grown at 28°C and 40°C (Figure 3). Relative to the wild-type strain, the expression of the *ctsR* gene was dramatically decreased in the mutants that lack a functional *ctsR* gene copy (161- to 984-fold), irrespective of the temperature of growth, confirming the integrity of the *ctsR* mutation in these strains (Table 1). Similarly, *hrcA* was decreased in the Δ*hrcA*::*cat*, and Δ*ctsR*Δ*hrcA*::*cat* mutants as compared to the wild type (145- to 241-fold; Table 1). The predicted HrcA and CtsR promoter binding motifs (*cis*-elements) [32,33] were used for MAST [49] analyses to predict the members of the HrcA and/or CtsR regulons, revealing several genes that appear to harbor the *cis*-acting motif of at least one of the transcription regulators (Table 1). Several of the CtsR regulon members that have previously been experimentally verified [31], were transcribed at higher levels in the Δ*ctsR* and Δ*ctsR*Δ*hrcA*::*cat* mutants grown at 28°C as compared to the wild-type, including *clpP*, *clpE*, *clpB*, *clpC*, *hsp1*, and *spx1* (Figure 3 and Table 1). In addition, a gene with unknown function (*lp*_*2061*) and an operon including 2 proteases (*hslU* and *hslV*) were expressed at elevated levels in the Δ*ctsR* strain. Of the predicted *hrcA* regulon members (Table 1), no altered expression pattern was detected for the *grpE*, *dnaK* and *dnaJ* genes, which are located in the same operon as *hrcA*, while *groEL* and *groES* expression patterns were increased in the Δ*hrcA*::*cat* mutant, at 28°C. A gene with unknown function (*lp*_*1880*) and an integrase/recombinase (*lp*_*1268*) were differentially expressed in the Δ*hrcA*::*cat* and Δ*ctsR*Δ*hrcA*::*cat* strains. Remarkably, the *hrcA* operon seems to have 2 CIRCE elements and a CtsR-targeted *cis*-element in its promoter region, which may suggest dual control of this regulon by both regulators. However, *hrcA* was not differentially expressed in the Δ*ctsR* mutant at control or elevated temperature. When identifying possible dually regulated genes, only *hsp1* had CtsR and HrcA *cis*-acting elements in the promoter region of this gene (Table 1), as was described previously [11]. This was supported by the upregulation of this gene in all three mutants compared to wild type at 28°C (Figure 3A and Table 1). It might be that sequence or position of the *cis*-acting elements matches with their expression level in the mutants. However, no significant correlation could be detected. Together this indicates that the deregulation of class I and/or class III stress responses by mutation of their regulators induces a partial alteration of expression of their (predicted) regulon members under the conditions tested. Besides the predicted regulon members, the transcription of genes classified to various functional categories appeared to be affected by *ctsR* and/or *hrcA* mutation, which will be discussed below.

### HrcA and CtsR mutation affect expression of genes encoding proteins with diverse functions

**Figure 4:**
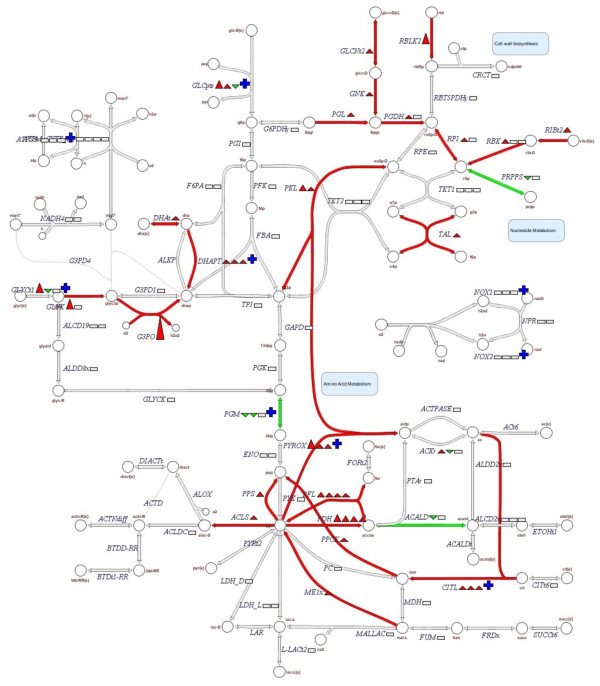
**Primary metabolic pathway of *L*. *plantarum *NZ3410 (Δ*ctsR*) compared to *L*. *plantarum *WCFS1 grown at 40°C.** Green lines or triangles indicate downregulation, whereas red lines or triangles indicate upregulation, open rectangles indicate no change, and plus symbols indicate that expression of more than 3 genes is acquired for enzyme production. Abbreviations are addressed in the Additional file 3: Table S2, according to Teusink *et al*. [28].

Additional genes coding for proteins from several functional categories were displaying altered transcription levels in the Δ*hrcA*::*cat* and Δ*ctsR* mutants as compared to the wild type. The *hrcA* mutation led to induced transcription of 29 transcription regulator encoding genes, including transcription regulators belonging to the AraC, LysR, MarR and TetR/AcrR family regulators. Several genes involved in primary metabolism were induced in the Δ*ctsR* strain compared to the wild type. These genes were involved in a variety of central metabolism reactions, centering around pyruvate dissipation and fermentation related reactions, including *pox*, *pfl*, *pdh*, *pps*, *mae*, *als*, and *cit* (Figure 4; abbreviations are addressed in the Additional file 3:Table S2). In addition, genes involved in pentose-5-phosphate pathway, producing D-xylulose-5-phosphate, which can be used for nucleotide synthesis or energy production, (including *xpkA*, *tkt1*, *deoM*, *rpiA1*, *gntK*, and *xfp*) were induced in the Δ*ctsR* strain compared to the wild type (Figure 4). Moreover, genes involved in sugar metabolism, such as *scrB* (sucrose), *pbg* (glucose), *lac* (galactose), *ara* (ribulose), and *iol* (inositol), were induced in this strain, as were genes involved in transport of other unspecified carbohydrate substrates and organic acids. These genes included sucrose (*pts26BCA*), glucose (*pts32*), maltodextrin (*mdx*, *msmX*), mannitol (*pts2A*), mannose (*lp*_*3643*, *pts9*), arabinose (*araP*), trehalose (*pts4ABC*) and sorbitol (*pts37A*, *pts38BC*) transporters. These results illustrate the impact of CtsR deregulation on the expression of metabolic genes, mainly affecting functions of primary carbohydrate import and central metabolic pathways, which was not observed in the *hrcA*-deficient strain. Nevertheless, the *hrcA*-mutation led to repression of genes involved in transport and binding functions, like those involved in transport of phosphate (*pst*), amino acids (*cho*, *sdA*, *lp*_*1722*, and *lp*_*3324*), and unknown substrates. Taken together these observations illustrate that deregulation of CtsR or HrcA elicits different response-profiles of transport and metabolism functions.

**Figure 5:**
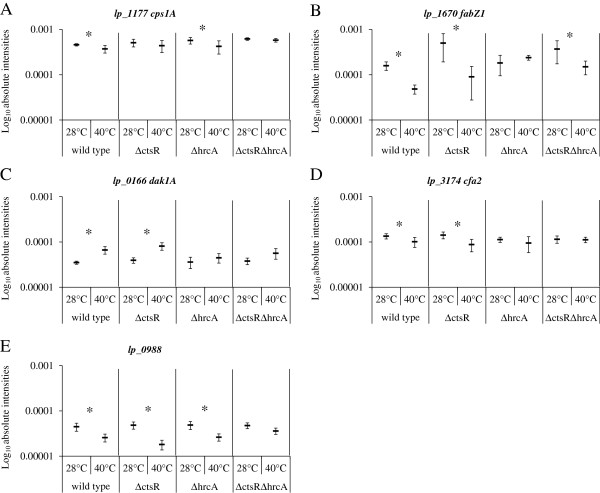
**Box plots displaying the absolute intensity of the first gene of the *cps *cluster *1 *(*lp*_*1177*; A), the *fab*-operon (*lp*_*1670*; B), *dak1A *(*lp*_*0166*; C), *cfa2 *(*lp*_*3174*; D), and *lp*_*0988 *(E) of *L*. *plantarum *WCFS1 (wild type), NZ3410 (Δ*ctsR*), NZ3425**^**CM **^**(Δ*hrcA*), and NZ3423**^**CM **^**(Δ*ctsR*Δ*hrcA*) grown at 28°C or 40°C.** Asterisk indicates that (part) of the loci are significant differentially expressed when compared to the strains growth at the other temperature.

In addition, the mutations of *hrcA* and/or *ctsR* appeared to play a role in the control of expression of some of the genes and functions that were affected by the temperature of growth in the wild-type strain (see above). Temperature-mediated regulation appeared to be (partially) lost in the Δ*ctsR* mutant (*cps1*), in the Δ*hrcA*::*cat* mutant (*fab* operon, *dak1A*, and *cfa2*), or in the Δ*ctsR*Δ*hrcA*::*cat* mutant [*lp*_*0988* (lipoprotein precursor), *cps1*, and *cfa2*] compared to that seen in the wild-type strain (Figure 5). This indicates that inactivation of both class I and III transcription regulation leads to deregulation of different combinations of cell envelope biosynthesis processes compared to deregulation of one of the regulators in a temperature-dependent way. Taken together, these findings indicate that some of the more prominent adaptations that the wild-type strain employs to combat elevated growth temperatures, appear to be deregulated in the HrcA and CtsR mutant strains.

### Combined HrcA and CtsR deficiency elicits pleiotropic deregulation of the stress control network

To characterize the gene-regulation consequences of the *hrcA* and *ctsR* single mutation relative to the double mutation, the significant regulatory profiles were reconstructed in gene-regulation networks for these strains relative to the wild-type strain at both 28°C (Figure 3A) and 40°C (Figure 3B). A relatively large number of genes displayed significant differential expression when comparing the Δ*ctsR*Δ*hrcA*::*cat* and wild type strains grown at either 28°C (513 genes) or 40°C (603 genes). At 28°C, these genes included almost all differentially expressed genes of the Δ*ctsR* and Δ*hrcA*::*cat* strains (Figure 3A). Conversely, less than one quarter and less than one third of the genes differentially expressed in the double mutant at 28°C were affected in the *ctsR* and *hrcA* single mutation at 40°C, respectively. Genes that are not differentially expressed in the other mutants than the Δ*ctsR* strain comprised for instance induction of energy metabolism (genes associated with TCA cycle, sugars, and glycolysis) and transport and binding proteins (e.g. the PTS system) and comprised 24 genes associated with regulatory functions for the Δ*hrcA*::*cat* strain. Overlapping genes of the *ctsR* or *hrcA* single mutation grown at 40°C with the double mutant grown at both temperatures included genes associated with the pentose phosphate pathway (*tkt1A* and *tkt1B*) and cell division (*ftsQ*, *parB1*, *parA*, and *parB2*), for the *ctsR* mutation and included genes associated with transport and binding proteins (e.g. ABC transporters and multidrug transporter proteins) for the *hrcA* mutation. In addition, genes associated with the cell envelope (such as genes encoding cell surface proteins and genes involved in fatty acid biosynthesis) were differentially expressed in all three mutants at 40°C. All three mutants affect temperature-independently the *dak1B* operon that is involved in glycerolipid metabolism. Moreover, approximately one third of the genes appeared to be consistently affected by the Δ*ctsR*Δ*hrcA*::*cat* mutation at both growth temperatures. The genes consistently affected by the Δ*ctsR*Δ*hrcA*::*cat* mutation included induction of genes associated with the cellular processes (such as cell division protein-encoding genes *ftsZ*, *ftsA*, and *ftsQ*), DNA metabolism (DNA ligase *ligA*, DNA helicase *pcrA*, and DNA-directed DNA polymerase I *polA*), transport and binding proteins (Na^+^/H^+^ antiporter *napA2*, mannose PTS *pts9D*, and 10 ABC transporters), and cell envelope remodeling (*cps*-cluster *1*, *fab*-locus, lipoprotein precursors *lp*_*1146* and *lp*_*1539*).

To further analyze the transcriptome profile of the Δ*ctsR*Δ*hrcA*::*cat* mutant grown at 28°C and 40°C, over-representative functional classes were identified (Figure 3). The BiNGO analysis tool was used to compare the Δ*ctsR*Δ*hrcA*::*cat* strain to the wild type, indicating that functional classes associated with cell envelope remodeling were induced, including the main class “cell envelope” with the sub-class “surface polysaccharides, lipopolysaccharides and antigens”, which were induced at both temperatures of growth. In addition, the main classes “cellular processes” and “DNA metabolism” were temperature-independently induced. Temperature specific cell envelope remodeling was also apparent from over-representation of the main class “fatty acid and phospholipid metabolism” when grown at 28°C, while several subclasses of cell surface proteins (“LPxTG anchored”, “membrane bound”, and “other”) were over-represented at 40°C. The main class “protein synthesis” was reduced in the *ctsR* and *hrcA* deficient strain only when grown at 40°C (Figure 3). Taken together, these data indicate that the cell employs highly adaptable, temperature-dependent systems involving many cell envelope associated functional classes to compensate for the absence of CtsR and HrcA regulation and that the expression of a large variety of additional genes appeared to be modulated compared to deregulation of one of the transcription factors.

### HrcA and/or CtsR are required for hydrogen peroxide resistance regulation in *L. plantarum*

Besides involvement of CtsR and HrcA to combat temperature stress, it is known that the transcription factors are associated with other stresses. To evaluate whether *ctsR* and/or *hrcA* may be involved in gastrointestinal (GI)-tract survival, the overlap between the differentially expressed genes in the constructed mutant and the genes identified as being induced in the murine intestine [52] were compared, revealing a substantial overlap (26%) with the genes that were induced in the *ctsR* deletion mutant compared to the wild type grown at 40°C. In addition, *L. plantarum* WCFS1 genes differentially expressed in response to porcine bile exposure [53], were also affected by the *ctsR* gene deletion when grown at 40°C (27%), albeit in the opposite direction. The possible role(s) of CtsR and/or HrcA in bile-stress response and tolerance was investigated by determination of the relative bile-tolerance of the three mutants relative to the wild type, revealing no significant role of either *ctsR* or *hrcA* in growth in the presence of bile (MRS containing 0.1% porcine bile; data not shown), suggesting that the *ctsR* and *hrcA* regulators do not play a role in bile tolerance. Although we cannot rule out the occurrence of polar effects that may have altered the expression some genes. In addition, the 3 mutant strains also displayed similar survival characteristics as the wild type in an *in vitro* assay that aims to mimic conditions encountered in the GI-tract [40]. Overall, these data suggest that although deregulation of CtsR and HrcA affects the expression of genes that were also differentially expressed under conditions relevant for the GI-tract, no experimental support could be found for a role of the *ctsR* and/or *hrcA* responses in survival under these conditions.

**Figure 6:**
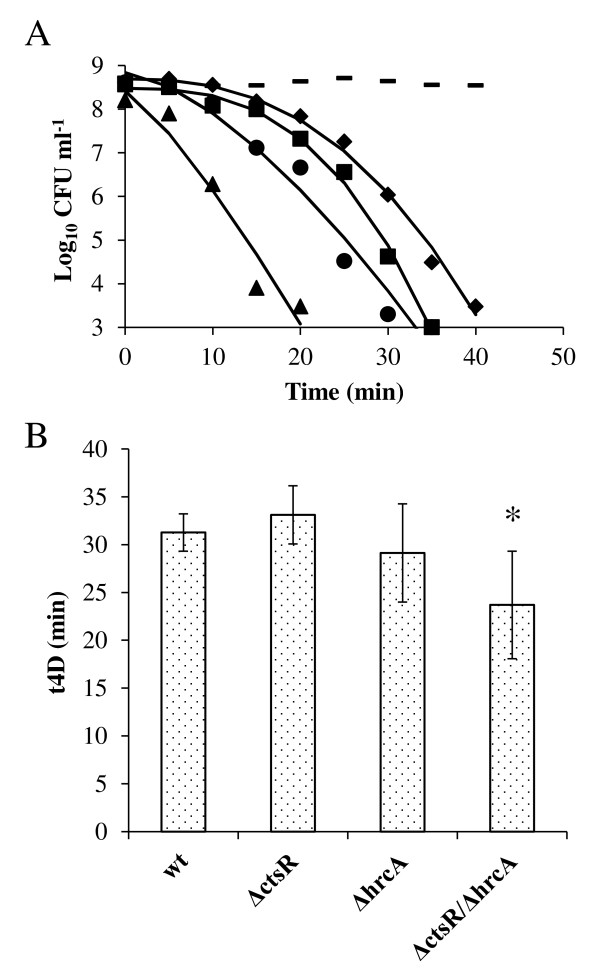
**Involvement of CtsR and HrcA in hydrogen peroxide resistance. A)** Colony forming units of *L. plantarum* WCFS1 (wt, squares), NZ3410 (Δ*ctsR*, diamonds), NZ3425^CM^ (Δ*hrcA*, circles), and NZ3423^CM^ (Δ*ctsR*Δ*hrcA*, triangles) cultures when subjected to 40 mM H_2_O_2_ exposure. As a control, the Δ*ctsR*Δ*hrcA* strain was taken for incubation in PBS without H_2_O_2_ (dashes). Lines indicate the fitted reparameterized Weibull model data. Data shown are representative for 3 independent experiments. **B)** Time to the first 4 log_10_ reductions (*t*_*4D*_) for the same strains as in panel A. The *t*_*4D*_-value is the parameter estimate obtained by fitting a reparameterized Weibull model through the data and average for the 4 experiments. Error bars represent the 95% confidence interval of the parameter estimate. Significant difference from the wt (*p* < 0.05) is indicated by *.

Another comparison between gene expression profiles of the Δ*ctsR*Δ*hrcA*::*cat* strain grown at 28°C and the response of *L. plantarum* to hydrogen peroxide [36], also revealed overlapping responses (21%). Analogous to what was observed for the bile responses (see above), the direction of gene expression changes were opposite for a number of genes affected both by H_2_O_2_ exposure, i.e., H_2_O_2_ induced expression of *lp*_*1163*, *dak1B*, *dak2*, *dak3*, *lp*_*1539*, the *cps1*-cluster and the Δ*ctsR*Δ*hrcA*::*cat* mutation elicited their repression. To evaluate the potential involvement of *ctsR* and *hrcA* in the oxidative-stress response and cognate tolerance towards H_2_O_2_ exposure, the wild type and mutant strains were grown to the exponential phase of growth (OD_600_ of 1.0) and their rate of loss of survival upon lethal H_2_O_2_ exposure (40 mM H_2_O_2_[54]), was followed over time by enumeration of colony forming units (Figure 6A). Compared to the wild-type strain, the Δ*ctsR* strain displayed similar rates of loss of survival, while the Δ*hrcA*::*cat* and especially the Δ*ctsR*Δ*hrcA*::*cat* strain were substantially reduced in their capability to tolerate H_2_O_2_ compared to the wild-type strain. This was already apparent after relatively short exposure to lethal peroxide stress levels, as is illustrated by the 10-fold reduced viability of the Δ*ctsR*Δ*hrcA*::*cat* strain after 10 min exposure to peroxide relative to the wild-type (Figure 6A). To quantitatively compare the data, a reparameterized Weibull model was fitted to the inactivation data according to Metselaar *et al*. [51]. In this adjusted Weibull model, the time to the first 4 decimal reductions (*t*_*4D*_) was calculated (Figure 6B). Shaping parameter *β* was comparable between the wild type and the variants, ranging from 2.20 to 3.42. The Δ*ctsR*Δ*hrcA*::*cat* strain showed a significant lower *t*_*4D*_ compared to the wild type (Figure 6B). In conclusion, these data underline that deregulation of the HrcA and CtsR regulons influences H_2_O_2_ tolerance.

## Discussion

In this paper, transcriptome profiles of *L. plantarum* WCFS1 were determined at reference and elevated temperatures. In the wild type strain, elevated temperature induced relatively major alterations in gene expression patterns. Many of these alterations suggest that adaptation of the cell envelope architecture is among the most important adaptive responses to elevated temperature. Relative to growth at 28°C, growth at 40°C induced the expression of several of the predicted CtsR and/or HrcA regulon members, e.g., *groES*, *groEL*, *clpP*, *clpB*, *clpE*, and *hsp1*[32,33]. This is in accordance with a study by Russo *et al*. that performed a global proteomic analysis of *L. plantarum* WCFS1 and a Δ*ctsR* mutant strain under optimal and heat stressed conditions [55]. Growth characteristics of the HrcA and CtsR deficient strains were considerably different from those of the wild-type, which was especially apparent from the mutants’ phenotype at 42°C. At this temperature, CtsR appeared to be required for maximum specific growth rates, while HrcA deletion increased colony forming capacity. Although the mechanism underlying the latter observation remains to be elucidated, it is most likely explained by culture-robustness heterogeneity, which in the *hrcA* deletion strain had shifted towards an average higher robustness level. While in several other organisms, *ctsR* mutation has been shown to enhance survival under stress conditions [56-59] this seemed not to be the case for *L. plantarum*, which is in agreement with previous studies in this organism [31]. Conversely, the enhanced colony forming capacity of the *hrcA* mutant at 42°C can be related to the deregulation of the class I stress response network, which is in agreement with the observation that similar mutations in other species enhanced their robustness under stress conditions [59,60]. However, in *Listeria monocytogenes*, *hrcA* deletion is suggested to be associated with increased heat sensitivity [61]. Overall, the impact of deregulation of the class I and class III stress responses on bacterial robustness is not very consistent and seems to vary considerably between species, which implies that extrapolation of the results obtained in specific species or strains to other organisms should be performed with great care.

To understand the HrcA and CtsR mediated stress adaptation, transcriptome analyses were performed comparing the transcriptional profiles of the HrcA- and CtsR-deficient strains at 28°C and 40°C. In addition, to unravel the intertwinement of the class I and class III stress response networks, a strain that lacked both repressors was included in this study. Transcriptome analyses of similar single mutants of either *hrcA* or *ctsR* have been reported for other species [4,62-66], and mutants lacking both repressors have been constructed in *Listeria monocytogenes*[62] and in *Staphylococcus aureus*[66]. Nevertheless, to the best of our knowledge, this study presents the first transcriptome analysis of a strain that is deficient for both regulators. Of the predicted *hrcA* regulon members, no altered expression pattern was detected for the *grpE*, *dnaK* and *dnaJ* genes, which may be due to the involvement of additional regulatory factors in the control of expression of this chaperone genecluster. For example, it has been demonstrated that carbon catabolite control mediated through CcpA can affect the expression of the *groELS* and *dnaK* operons in *L. plantarum*, and that in a CcpA-deficient strain the expression of these functions could not be fully induced leading to reduced stress tolerance levels. Although these observations may not completely explain the lack of activation of *dnaK* operon expression in the *hrcA* mutant, they clearly imply that CcpA-activation could contribute to expression of the *dnaK* operon [67,68]. Moreover, in other organisms, e.g. in *Streptococcus pneumonia*, the transcription of the *dnaK* and *groEL* operons is regulated by the medium concentration of Ca^2+^ as well as by HrcA [69], suggesting that additional environmental factors may modulate *hrcA* regulation of specific target genes and operons of its regulon. Although *lp*_*0726* is a predicted *hrcA* regulon member, its transcription level was increased in the Δ*ctsR* and Δ*ctsR*Δ*hrcA*::*cat* mutants. Besides transcriptional changes in the predicted regulons, *hrcA* and *ctsR* mutation led to a differential expression of genes involved in many functional classes during control and elevated temperature.

One of the deteriorating consequences encountered by cells growing at temperatures that can be considered as stress temperatures is denaturation and aggregation of proteins [70]. Lack of appropriate control of both the protein folding support (chaperones) and protein quality (Clp proteolysis) may elicit complementing gene expression responses involving genes belonging to different functional classes and affecting numerous cellular processes. These responses may include altered levels of regulator proteins in the cell, which may elicit changes in expression of a variety of regulons. Moreover, the levels of regulator protein may be differentially affected by the temperature of growth, leading to temperature-specific response of various regulatory networks, as was observed in this study. The drastic transcriptome changes elicited in the strain that lacks both CtsR and HrcA at control temperature is illustrative for the magnitude and complexity of the response required for the compensation for the deregulation of both class I and III stress responses. In addition, the results pinpoint that cell envelope remodeling plays an important role in the temperature adaptation in the wild-type strain, but is also prominently affected by the disruption of class I and III stress response networks. Intriguingly, it has been proposed that in prokaryotes heat shock responses are predominantly controlled by the membrane physical state [71-73], which is in agreement with the finding that adaptive responses include many membrane and envelope modulating functions. Moreover, HrcA has been proposed to be a membrane-associated protein in *Helicobacter pylori*, and even an integral membrane protein in *Streptococcus pneumoniae*. In addition, the *hrcA*-regulon member GroELS of *Escherichia coli* is involved in folding of both soluble and membrane-associated proteins, while concomitantly stabilizing lipid membranes [49,74,75].

To understand the role of HrcA and CtsR in other stress conditions besides elevated temperature, the deregulation responses in the *hrcA* and *ctsR* mutant strains were compared with responses in the wild-type *L. plantarum* strain upon its exposure to specific stress conditions. The mutant lacking both *ctsR* and *hrcA* displayed significant decreased H_2_O_2_ tolerance levels compared with the wild type, suggesting that appropriate classI and III stress-regulation are required for optimal peroxide stress adaptation in *L. plantarum*. Downregulation of genes encoding proteins involved in membrane lipid synthesis (*dak1B*, *dak2*, *dak3*, and *lp*_*1539*) and cell wall (*cps1* cluster) in this mutant possibly induce cell envelope modifications that weaken the cell when exposed to peroxide stress. Furthermore, class I and class III stress responses were previously reported to be involved in oxidative stress tolerance in *Fusobacterium nucleatum*, which was associated to induction of ClpB and DnaK in response to H_2_O_2_ stress [76]. A potentially more indirect link may exist between the Clp protease and H_2_O_2_ stress responses in *B. subtilis*, where Clp protease activity is involved in regulation of Spx [21], which in its turn was shown to be induced upon H_2_O_2_ exposure [77].

Overall, deregulation of the CtsR and HrcA regulons in *L. plantarum* elicits compensatory responses that can be characterized by differential transcriptome analyses. These analyses reveal the modulation of several major functional classes, which appears to be temperature-dependent. Therefore, proper control of the CtsR and HrcA regulons are essential for maintaining optimal cell function in changing environments. Moreover, gene regulatory network reconstructions are essential to survey the full regulatory response of an organism. In these networks, the role of the canonical class I and III stress response regulators will be of great importance, because of their pleiotropic character.

## Competing interests

The authors declare that they have no competing interests.

## Authors’ contributions

HvB-vdV, MW, PAB, and MK designed the experiments, HvB-vdV and RB performed the experiments, HvB-vdV, RB, MW, PAB, and MK interpreted the data, and HvB-vdV, PAB, and MK drafted and revised the manuscript. All authors approved the version of the manuscript to be published.

## Supplementary Material

Additional file 1: Figure S1 Hybridization scheme for DNA microarrays using cDNA derived from *L. plantarum* WCFS1 (WT), NZ3410 (Δ*ctsR*; dC), NZ3425CM (Δ*hrcA::cat*; dH), and NZ3423CM (Δ*ctsR*Δ*hrcA::cat*; dCdH). Temperature in C° is indicated after the slash. Duplicates were included (between brackets) and circled number indicates hybridization number. Tail and head of the arrow represent Cy3 and Cy5 labeling, respectively. Click here for file

Additional file 2: Table S1 Differentially regulated genes in *L. plantarum* WCFS1 grown at 40°C compared to 28°C. Click here for file

Additional file 3: Table S2 Abbreviations used in Figure 4. Click here for file

## References

[B1] Li H Cao Y Lactic acid bacterial cell factories for gamma-aminobutyric acid Amino Acids 2010 39 5 1107 1116 10.1007/s00726-010-0582-7 20364279

[B2] Bourdichon F Casaregola S Farrokh C Frisvad JC Gerds ML Hammes WP Harnett J Huys G Laulund S Ouwehand A Food fermentations: microorganisms with technological beneficial use Int J Food Microbiol 2012 154 3 87 97 10.1016/j.ijfoodmicro.2011.12.030 22257932

[B3] Ahrne S Nobaek S Jeppsson B Adlerberth I Wold AE Molin G The normal *Lactobacillus* flora of healthy human rectal and oral mucosa J Appl Microbiol 1998 85 1 88 94 10.1046/j.1365-2672.1998.00480.x 9721659

[B4] Lee NK Yun CW Kim SW Chang HI Kang CW Paik HD Screening of Lactobacilli derived from chicken feces and partial characterization of *Lactobacillus acidophilus* A12 as an animal probiotics J Microbiol Biotechnol 2008 18 2 338 342 18309281

[B5] Siezen RJ Tzeneva VA Castioni A Wels M Phan HT Rademaker JL Starrenburg MJ Kleerebezem M Molenaar D Van Hylckama Vlieg JE Phenotypic and genomic diversity of *Lactobacillus plantarum* strains isolated from various environmental niches Environ Microbiol 2010 12 3 758 773 10.1111/j.1462-2920.2009.02119.x 20002138

[B6] FAO/WHO Evaluation of Health and Nutritional Properties of Powder Milk with Live Lactic Acid Bacteria Report of FAO/WHO expert consultation 1–4 October 2001

[B7] Lebeer S Vanderleyden J De Keersmaecker SC Host interactions of probiotic bacterial surface molecules: comparison with commensals and pathogens Nat Rev Microbiol 2010 8 3 171 184 10.1038/nrmicro2297 20157338

[B8] Kleerebezem M Hols P Bernard E Rolain T Zhou M Siezen RJ Bron PA The extracellular biology of the lactobacilli FEMS Microbiol Rev 2010 34 2 199 230 10.1111/j.1574-6976.2009.00208.x 20088967

[B9] Van de Guchte M Serror P Chervaux C Smokvina T Ehrlich SD Maguin E Stress responses in lactic acid bacteria Antonie Van Leeuwenhoek 2002 82 1–4 187 216 12369188

[B10] De Angelis M Gobbetti M Environmental stress responses in *lactobacillus*: a review Proteomics 2004 4 1 106 122 10.1002/pmic.200300497 14730676

[B11] Spano G Massa S Environmental stress response in wine lactic acid bacteria: beyond *bacillus subtilis* Crit Rev Microbiol 2006 32 2 77 86 10.1080/10408410600709800 16809231

[B12] Mills S Stanton C Fitzgerald G Ross RP Enhancing the stress responses of probiotics for a lifestyle from gut to product and back again Microb Cell Fact 2011 10 Suppl 1 15 10.1186/1475-2859-10-S1-S15 21995734 PMC3231925

[B13] Schumann W The *Bacillus subtilis* heat shock stimulon Cell Stress Chaperones 2003 8 3 207 217 10.1379/1466-1268(2003)008<0207:TBSHSS>2.0.CO;2 14984053 PMC514873

[B14] Darmon E Noone D Masson A Bron S Kuipers OP Devine KM van Dijl JM A novel class of heat and secretion stress-responsive genes is controlled by the autoregulated CssRS two-component system of *Bacillus subtilis* J Bacteriol 2002 184 20 5661 5671 10.1128/JB.184.20.5661-5671.2002 12270824 PMC139597

[B15] Helmann JD Wu MF Kobel PA Gamo FJ Wilson M Morshedi MM Navre M Paddon C Global transcriptional response of *Bacillus subtilis* to heat shock J Bacteriol 2001 183 24 7318 7328 10.1128/JB.183.24.7318-7328.2001 11717291 PMC95581

[B16] Narberhaus F Negative regulation of bacterial heat shock genes Mol Microbiol 1999 31 1 1 8 10.1046/j.1365-2958.1999.01166.x 9987104

[B17] Corcoran BM Stanton C Fitzgerald G Ross RP Life under stress: the probiotic stress response and how it may be manipulated Curr Pharm Des 2008 14 14 1382 1399 10.2174/138161208784480225 18537661

[B18] Van Bokhorst-van de Veen H Bron PA Wels M Kleerebezem M Tsakalidou E, Papadimitriou K Engineering robust lactic acid bacteria Stress Responses of Lactic Acid Bacteria 2011 US: Springer 369 394

[B19] Elsholz AK Gerth U Hecker M Regulation of CtsR activity in low GC, Gram + bacteria Adv Microb Physiol 2010 57 119 144 21078442 10.1016/B978-0-12-381045-8.00003-5

[B20] Derre I Rapoport G Msadek T CtsR, a novel regulator of stress and heat shock response, controls *clp* and molecular chaperone gene expression in Gram-positive bacteria Mol Microbiol 1999 31 1 117 131 10.1046/j.1365-2958.1999.01152.x 9987115

[B21] Frees D Savijoki K Varmanen P Ingmer H Clp ATPases and ClpP proteolytic complexes regulate vital biological processes in low GC, Gram-positive bacteria Mol Microbiol 2007 63 5 1285 1295 10.1111/j.1365-2958.2007.05598.x 17302811

[B22] Chastanet A Msadek T ClpP of *Streptococcus salivarius* is a novel member of the dually regulated class of stress response genes in Gram-positive bacteria J Bacteriol 2003 185 2 683 687 10.1128/JB.185.2.683-687.2003 12511518 PMC145324

[B23] Marco ML Pavan S Kleerebezem M Towards understanding molecular modes of probiotic action Curr Opin Biotechnol 2006 17 2 204 210 10.1016/j.copbio.2006.02.005 16510275

[B24] Vesa T Pochart P Marteau P Pharmacokinetics of *Lactobacillus plantarum* NCIMB 8826, *Lactobacillus fermentum* KLD, and *Lactococcus lactis* MG 1363 in the human gastrointestinal tract Aliment Pharmacol Ther 2000 14 6 823 828 10.1046/j.1365-2036.2000.00763.x 10848668

[B25] Van Bokhorst-van de Veen H van Swam I Wels M Bron PA Kleerebezem M Congruent strain specific intestinal persistence of *Lactobacillus plantarum* in an intestine-mimicking *in vitro* system and in human volunteers PLoS ONE 2012 7 9 44588 10.1371/journal.pone.0044588 PMC3435264 22970257

[B26] Kleerebezem M Boekhorst J van Kranenburg R Molenaar D Kuipers OP Leer R Tarchini R Peters SA Sandbrink HM Fiers MW Complete genome sequence of *Lactobacillus plantarum* WCFS1 Proc Natl Acad Sci USA 2003 100 4 1990 1995 10.1073/pnas.0337704100 12566566 PMC149946

[B27] Teusink B Van Enckevort FH Francke C Wiersma A Wegkamp A Smid EJ Siezen RJ *In silico* reconstruction of the metabolic pathways of *Lactobacillus plantarum*: comparing predictions of nutrient requirements with those from growth experiments Appl Environ Microbiol 2005 71 11 7253 7262 10.1128/AEM.71.11.7253-7262.2005 16269766 PMC1287688

[B28] Teusink B Wiersma A Molenaar D Francke C de Vos WM Siezen RJ Smid EJ Analysis of growth of *Lactobacillus plantarum* WCFS1 on a complex medium using a genome-scale metabolic model J Biol Chem 2006 281 52 40041 40048 10.1074/jbc.M606263200 17062565

[B29] Lambert JM Bongers RS Kleerebezem M Cre-*lox*-based system for multiple gene deletions and selectable-marker removal in *Lactobacillus plantarum* Appl Environ Microbiol 2007 73 4 1126 1135 10.1128/AEM.01473-06 17142375 PMC1828656

[B30] Fiocco D Collins M Muscariello L Hols P Kleerebezem M Msadek T Spano G The *Lactobacillus plantarum ftsH* gene is a novel member of the CtsR stress response regulon J Bacteriol 2009 191 5 1688 1694 10.1128/JB.01551-08 19074391 PMC2648225

[B31] Fiocco D Capozzi V Collins M Gallone A Hols P Guzzo J Weidmann S Rieu A Msadek T Spano G Characterization of the CtsR stress response regulon in *Lactobacillus plantarum* J Bacteriol 2010 192 3 896 900 10.1128/JB.01122-09 19933364 PMC2812460

[B32] Wels M Francke C Kerkhoven R Kleerebezem M Siezen RJ Predicting *cis*-acting elements of *Lactobacillus plantarum* by comparative genomics with different taxonomic subgroups Nucleic Acids Res 2006 34 7 1947 1958 10.1093/nar/gkl138 16614445 PMC1435977

[B33] Wels M Overmars L Francke C Kleerebezem M Siezen RJ Reconstruction of the regulatory network of *Lactobacillus plantarum* WCFS1 on basis of correlated gene expression and conserved regulatory motifs Microb Biotechnol 2011 4 3 333 344 10.1111/j.1751-7915.2010.00217.x 21375715 PMC3818992

[B34] Pieterse B Leer RJ Schuren FH Van der Werf MJ Unravelling the multiple effects of lactic acid stress on *Lactobacillus plantarum* by transcription profiling Microbiology 2005 151 Pt 12 3881 3894 16339934 10.1099/mic.0.28304-0

[B35] Serrano LM Molenaar D Wels M Teusink B Bron PA De Vos WM Smid EJ Thioredoxin reductase is a key factor in the oxidative stress response of *Lactobacillus plantarum* WCFS1 Microb Cell Fact 2007 6 1 29 10.1186/1475-2859-6-29 17725816 PMC2174512

[B36] Stevens MJA Transcriptiome Response of Lactobacillus Plantarum to Global Regulator Deficiency, Stress and other Environmental Conditions 2008 Wageningen: Thesis Wageningen University

[B37] Fiocco D Capozzi V Goffin P Hols P Spano G Improved adaptation to heat, cold, and solvent tolerance in *Lactobacillus plantarum* Appl Microbiol Biotechnol 2007 77 4 6 10.1007/s00253-007-1228-x 17960374

[B38] Van Bokhorst-van De Veen H Abee T Tempelaars M Bron PA Kleerebezem M Marco ML Short- and long-term adaptation to ethanol stress and its cross-protective consequences in *Lactobacillus plantarum* Appl Environ Microbio 2011 77 15 5247 5256 10.1128/AEM.00515-11 PMC3147428 21705551

[B39] Bron PA Meijer M Bongers RS De Vos WM Kleerebezem M Dynamics of competitive population abundance of *Lactobacillus plantarum ivi* gene mutants in faecal samples after passage through the gastrointestinal tract of mice J Appl Microbiol 2007 103 5 1424 1434 10.1111/j.1365-2672.2007.03376.x 17953553

[B40] Van Bokhorst-van De Veen H Lee IC Marco ML Wels M Bron PA Kleerebezem M Modulation of *Lactobacillus plantarum* gastrointestinal robustness by fermentation conditions enables identification of bacterial robustness markers PLoS ONE 2012 7 7 e39053 10.1371/journal.pone.0039053 22802934 PMC3389004

[B41] Meijerink M van Hemert S Taverne N Wels M de Vos P Bron PA Savelkoul HF van Bilsen J Kleerebezem M Wells JM Identification of genetic loci in *Lactobacillus plantarum* that modulate the immune response of dendritic cells using comparative genome hybridization PLoS ONE 2010 5 5 e10632 10.1371/journal.pone.0010632 20498715 PMC2869364

[B42] Marco ML Peters TH Bongers RS Molenaar D Van Hemert S Sonnenburg JL Gordon JI Kleerebezem M Lifestyle of *Lactobacillus plantarum* in the mouse caecum Environ Microbiol 2009 11 10 2747 2757 10.1111/j.1462-2920.2009.02001.x 19638173 PMC2978903

[B43] Yang YH Dudoit S Luu P Lin DM Peng V Ngai J Speed TP Normalization for cDNA microarray data: a robust composite method addressing single and multiple slide systematic variation Nucleic Acids Res 2002 30 4 e15 10.1093/nar/30.4.e15 11842121 PMC100354

[B44] Kuipers OP Kok J Trelles O Garcia de la Nava J Van Hijum SA MicroPreP: a cDNA microarray data pre-processing framework Appl Bioinformatics 2003 2 4 241 244 15130795

[B45] Baldi P Long AD A Bayesian framework for the analysis of microarray expression data: regularized t -test and statistical inferences of gene changes Bioinformatics 2001 17 6 509 519 10.1093/bioinformatics/17.6.509 11395427

[B46] Shannon P Markiel A Ozier O Baliga NS Wang JT Ramage D Amin N Schwikowski B Ideker T Cytoscape: a software environment for integrated models of biomolecular interaction networks Genome Res 2003 13 11 2498 2504 10.1101/gr.1239303 14597658 PMC403769

[B47] Maere S Heymans K Kuiper M BiNGO: a Cytoscape plugin to assess overrepresentation of gene ontology categories in biological networks Bioinformatics 2005 21 16 3448 3449 10.1093/bioinformatics/bti551 15972284

[B48] Bailey TL Boden M Buske FA Frith M Grant CE Clementi L Ren J Li WW Noble WS MEME SUITE: tools for motif discovery and searching Nucleic Acids Res 2009 37 Web Server issue 202 208 10.1093/nar/gkp335 PMC2703892 19458158

[B49] Torok Z Horvath I Goloubinoff P Kovacs E Glatz A Balogh G Vigh L Evidence for a lipochaperonin: association of active protein-folding GroESL oligomers with lipids can stabilize membranes under heat shock conditions Proc Natl Acad Sci USA 1997 94 6 2192 2197 10.1073/pnas.94.6.2192 9122170 PMC20063

[B50] Bron PA Marco M Hoffer SM Van Mullekom E De Vos WM Kleerebezem M Genetic characterization of the bile salt response in *Lactobacillus plantarum* and analysis of responsive promoters *in vitro* and *in situ* in the gastrointestinal tract J Bacteriol 2004 186 23 7829 7835 10.1128/JB.186.23.7829-7835.2004 15547253 PMC529069

[B51] Metselaar KI Den Besten HM Abee T Moezelaar R Zwietering MH Isolation and quantification of highly acid resistant variants of *Listeria monocytogenes* Int J Food Microbiol 2013 166 3 508 514 10.1016/j.ijfoodmicro.2013.08.011 24051176

[B52] Bron PA Grangette C Mercenier A De Vos WM Kleerebezem M Identification of *Lactobacillus plantarum* genes that are induced in the gastrointestinal tract of mice J Bacteriol 2004 186 17 5721 5729 10.1128/JB.186.17.5721-5729.2004 15317777 PMC516819

[B53] Bron PA Molenaar D De Vos WM Kleerebezem M DNA micro-array-based identification of bile-responsive genes in *Lactobacillus plantarum* J Appl Microbiol 2006 100 4 728 738 10.1111/j.1365-2672.2006.02891.x 16553727

[B54] Stevens MJ Molenaar D De Jong A De Vos WM Kleerebezem M Involvement of the mannose phosphotransferase system of *Lactobacillus plantarum* WCFS1 in peroxide stress tolerance Appl Environ Microbiol 2010 76 11 3748 3752 10.1128/AEM.00073-10 20363783 PMC2876449

[B55] Russo P De la Luz MM Capozzi V De Palencia PF Lopez P Spano G Fiocco D Comparative proteomic analysis of *Lactobacillus plantarum* WCFS1 and delta*ctsR* mutant strains under physiological and heat stress conditions Int J Mol Sci 2012 13 9 10680 10696 23109816 10.3390/ijms130910680 PMC3472708

[B56] Hufner E Markieton T Chaillou S Crutz-Le Coq AM Zagorec M Hertel C Identification of *Lactobacillus sakei* genes induced during meat fermentation and their role in survival and growth Appl Environ Microbiol 2007 73 8 2522 2531 10.1128/AEM.02396-06 17308175 PMC1855608

[B57] Nair S Derre I Msadek T Gaillot O Berche P CtsR controls class III heat shock gene expression in the human pathogen *Listeria monocytogenes* Mol Microbiol 2000 35 4 800 811 10.1046/j.1365-2958.2000.01752.x 10692157

[B58] Karatzas KA Bennik MH Characterization of a *Listeria monocytogenes* Scott A isolate with high tolerance towards high hydrostatic pressure Appl Environ Microbiol 2002 68 7 3183 3189 10.1128/AEM.68.7.3183-3189.2002 12088993 PMC126791

[B59] Zotta T Asterinou K Rossano R Ricciardi A Varcamonti M Parente E Effect of inactivation of stress response regulators on the growth and survival of *Streptococcus thermophilus* Sfi39 Int J Food Microbiol 2009 129 3 211 220 10.1016/j.ijfoodmicro.2008.11.024 19128851

[B60] Schulz A Schumann W *hrcA*, the first gene of the *Bacillus subtilis* dnaK operon encodes a negative regulator of class I heat shock genes J Bacteriol 1996 178 4 1088 1093 8576042 10.1128/jb.178.4.1088-1093.1996 PMC177769

[B61] Hu Y Oliver HF Raengpradub S Palmer ME Orsi RH Wiedmann M Boor KJ Transcriptomic and phenotypic analyses suggest a network between the transcriptional regulators HrcA and sigmaB in *Listeria monocytogenes* Appl Environ Microbiol 2007 73 24 7981 7991 10.1128/AEM.01281-07 17965207 PMC2168140

[B62] Elsholz AK Hempel K Pother DC Becher D Hecker M Gerth U CtsR inactivation during thiol-specific stress in low GC, Gram + bacteria Mol Microbiol 2011 79 3 772 785 10.1111/j.1365-2958.2010.07489.x 21208299

[B63] Van Baarlen P Troost F van der Meer C Hooiveld G Boekschoten M Brummer RJ Kleerebezem M Human mucosal *in vivo* transcriptome responses to three lactobacilli indicate how probiotics may modulate human cellular pathways Proc Natl Acad Sci USA 2011 108 Suppl 1 4562 4569 20823239 10.1073/pnas.1000079107 PMC3063594

[B64] Hummelen R Vos AP Van't Land B Van Norren K Reid G Altered host-microbe interaction in HIV: a target for intervention with pro- and prebiotics Int Rev Immunol 2010 29 5 485 513 10.3109/08830185.2010.505310 20839912

[B65] Roncarati D Danielli A Spohn G Delany I Scarlato V Transcriptional regulation of stress response and motility functions in *Helicobacter pylori* is mediated by HspR and HrcA J Bacteriol 2007 189 20 7234 7243 10.1128/JB.00626-07 17693507 PMC2168435

[B66] Chastanet A Fert J Msadek T Comparative genomics reveal novel heat shock regulatory mechanisms in *Staphylococcus aureus* and other Gram-positive bacteria Mol Microbiol 2003 47 4 1061 1073 10.1046/j.1365-2958.2003.03355.x 12581359

[B67] Castaldo C Siciliano RA Muscariello L Marasco R Sacco M CcpA affects expression of the *groESL* and *dnaK* operons in *Lactobacillus plantarum* Microb Cell Fact 2006 5 35 10.1186/1475-2859-5-35 17129387 PMC1676014

[B68] Mazzeo MF Cacace G Peluso A Zotta T Muscariello L Vastano V Parente E Siciliano RA Effect of inactivation of *ccpA* and aerobic growth in *lactobacillus plantarum*: a proteomic perspective J Proteomics 2012 75 13 4050 4061 10.1016/j.jprot.2012.05.019 22634038

[B69] Kim SN Bae YG Rhee DK Dual regulation of *dnaK* and *groE* operons by HrcA and Ca++ in *Streptococcus pneumoniae* Arch Pharm Res 2008 31 4 462 467 10.1007/s12272-001-1179-4 18449503

[B70] Somero GN Proteins and temperature Annu Rev Physiol 1995 57 43 68 10.1146/annurev.ph.57.030195.000355 7778874

[B71] Porta A Torok Z Horvath I Franceschelli S Vigh L Maresca B Genetic modification of the *Salmonella* membrane physical state alters the pattern of heat shock response J Bacteriol 2010 192 7 1988 1998 10.1128/JB.00988-09 20139186 PMC2838041

[B72] Porta A Eletto A Torok Z Franceschelli S Glatz A Vigh L Maresca B Changes in membrane fluid state and heat shock response cause attenuation of virulence J Bacteriol 2010 192 7 1999 2005 10.1128/JB.00990-09 20139193 PMC2838053

[B73] Coucheney F Gal L Beney L Lherminier J Gervais P Guzzo J A small HSP, Lo18, interacts with the cell membrane and modulates lipid physical state under heat shock conditions in a lactic acid bacterium Biochim Biophys Acta 2005 1720 1–2 92 98 16472556 10.1016/j.bbamem.2005.11.017

[B74] Kwon HY Kim EH Tran TD Pyo SN Rhee DK Reduction-sensitive and cysteine residue-mediated *Streptococcus pneumoniae* HrcA oligomerization *in vitro* Mol Cells 2009 27 2 149 157 10.1007/s10059-009-0019-x 19277496

[B75] Roncarati D Spohn G Tango N Danielli A Delany I Scarlato V Expression, purification and characterization of the membrane-associated HrcA repressor protein of Helicobacter pylori Protein Expr Purif 2007 51 2 267 275 10.1016/j.pep.2006.08.002 16997572

[B76] Steeves CH Potrykus J Barnett DA Bearne SL Oxidative stress response in the opportunistic oral pathogen *Fusobacterium nucleatum* Proteomics 2011 11 10 2027 2037 10.1002/pmic.201000631 21563313

[B77] Tam LT Antelmann H Eymann C Albrecht D Bernhardt J Hecker M Proteome signatures for stress and starvation in *Bacillus subtilis* as revealed by a 2-D gel image color coding approach Proteomics 2006 6 16 4565 4585 10.1002/pmic.200600100 16847875

