# Supplementary material for: Transcriptome signatures of class I and III stress response deregulation in Lactobacillus plantarum reveal pleiotropic adaptation
Source: Microb Cell Fact. 2013 Nov 18;12:112. doi: 10.1186/1475-2859-12-112 (PMC3842655; doi:10.1186/1475-2859-12-112)

Supplementary information related to:

Transcriptome signatures of class I and III stress response deregulation in *Lactobacillus plantarum* reveal pleiotropic adaptation

Running title: *ctsR* and *hrcA* deregulation in *L. plantarum* WCFS1

Hermien van Bokhorst-van de Veen, Roger S. Bongers, Michiel Wels, Peter A. Bron, and Michiel Kleerebezem

Supplementary Figure S1

Hybridization scheme for DNA microarrays using cDNA derived from *L. plantarum* WCFS1 (WT), NZ3410 (Δ*ctsR*; dC), NZ3425^{CM} (Δ*hrcA::cat*; dH), and NZ3423^{CM} (Δ*ctsR*Δ*hrcA::cat*; dCdH). Temperature in C° is indicated after the slash. Duplicates were included (between brackets) and circled number indicates hybridization number. Tail and head of the arrow represent Cy3 and Cy5 labeling, respectively.

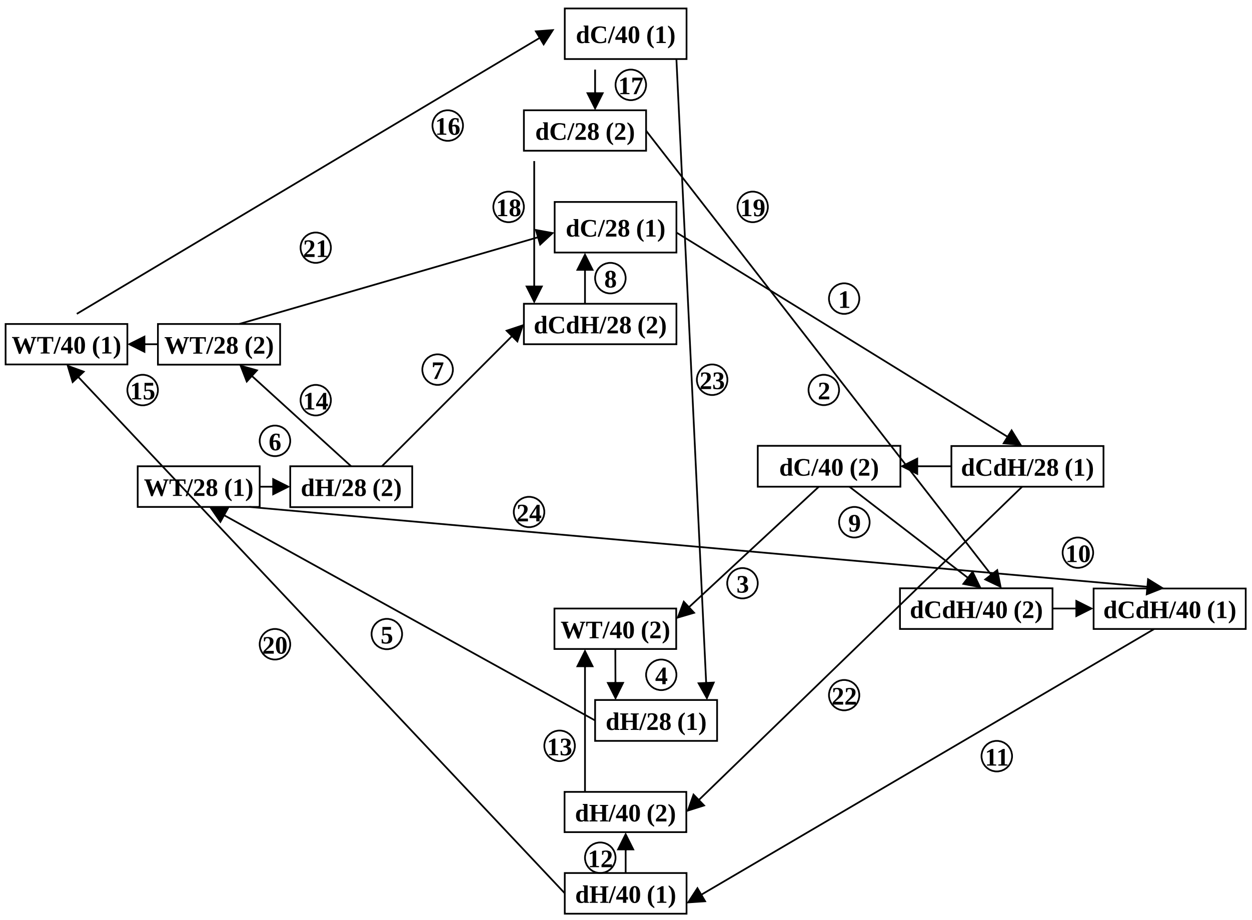

Supplement: Additional file 1: Figure S1 — Hybridization scheme for DNA microarrays using cDNA derived from L. plantarum WCFS1 (WT), NZ3410 (ΔctsR; dC), NZ3425CM (ΔhrcA::cat; dH), and NZ3423CM (ΔctsRΔhrcA::cat; dCdH). Temperature in C° is indicated after the slash. Duplicates were included (between brackets) and circled number indicates hybridization number. Tail and head of the arrow represent Cy3 and Cy5 labeling, respectively. [file 1475-2859-12-112-S1.pdf]
