# Supplementary material for: Transcriptome signatures of class I and III stress response deregulation in Lactobacillus plantarum reveal pleiotropic adaptation
Source: Microb Cell Fact. 2013 Nov 18;12:112. doi: 10.1186/1475-2859-12-112 (PMC3842655; doi:10.1186/1475-2859-12-112)
Supplement: Additional file 2: Table S1 — Differentially regulated genes in L. plantarum WCFS1 grown at 40°C compared to 28°C. [file 1475-2859-12-112-S2.pdf]

Supplementary information related to:

Transcriptome signatures of class I and III stress response deregulation in *Lactobacillus plantarum* reveal pleiotropic adaptation

Running title: *ctsR* and *hrcA* deregulation in *L. plantarum* WCFS1

Hermien van Bokhorst-van de Veen, Roger S. Bongers, Michiel Wels, Peter A. Bron, and Michiel Kleerebezem

Supplementary Table S1. Differentially regulated genes in *L. plantarum* WCFS1 grown at 40°C compared to 28°C.

Locus	Name	Function	Fold-change (40°C over 28°C)[a]	p-value[b]
lp_1085	aroA	phospho-2-dehydro-3-deoxyheptonate aldolase / chorismate mutase	0.491388	0.001534
lp_1654	trpD	anthranilate phosphoribosyltransferase	0.20588	8.92E-05
lp_3283	lp_3283	methonine synthase (cobalamine-independent), C-terminal domain (putative)	0.22902	0.000469
lp_0528	argC2	N-acetyl-gamma-glutamyl-phosphate reductase	0.475302	0.016291
lp_3085	asnB2	asparagine synthase (glutamine-hydrolysing)	0.473273	5.52E-10
lp_2553	hisI	phosphoribosyl-AMP cyclohydrolase	0.333045	0.011242
lp_0571	hom2	homoserine dehydrogenase	0.481099	9.46E-09
lp_0572	thrB	homoserine kinase	0.390696	1.44E-12
lp_1296	hemH	ferrochelatase	0.214405	2.13E-09
lp_1546	lp_1546	prenyltransferase	0.475679	1.75E-06
lp_1491	mobA	molybdopterin-guanine dinucleotide biosynthesis protein MobA (putative)	0.060745	1.28E-05
lp_1494	moeA	molybdopterin biosynthesis protein MoeA	0.473006	0.002091
lp_0113	thiM	hydroxyethylthiazole kinase	0.414601	0.000172
lp_1470	csd1	cysteine desulfurase	0.311174	2.45E-08
lp_2620	lp_2620	cell surface hydrolase, membrane-bound	0.480333	0.00017
lp_2911	lp_2911	membrane-bound protease, CAAX family	0.414687	4.85E-08
lp_0297	lp_0297	extracellular protein	0.366696	1.02E-09
lp_1446	lp_1446	extracellular protein	0.39904	0.04687
lp_3067a	lp_3067a	extracellular protein, C-terminal part	0.116892	0.001784
lp_3178	lp_3178	extracellular protein (putative)	0.219701	2.49E-06
lp_3015	lp_3015	extracellular transglycosylase (putative)	0.348796	3.03E-10
lp_0107	larC2	lactate racemization operon protein LarC, C-terminal domain	0.303601	0.002058
lp_2361	murA2	UDP-N-acetylglucosamine 1-carboxyvinyltransferase	0.498503	2.35E-08
lp_3254	lrgA	murein hydrolase export protein (putative)	0.271242	5.45E-07
lp_3255	lrgB	murein hydrolase regulator (putative)	0.404476	1.92E-08
lp_0844	licD	lipooligosaccharide cholinephosphotransferase (putative)	0.446015	9.53E-05
lp_1215	cps3A	glycosyltransferase	0.420076	2.58E-05
lp_1220	cps3D	polysaccharide biosynthesis protein (putative)	0.175414	8.18E-13
lp_1221	cps3E	polysaccharide biosynthesis protein (putative)	0.343674	2.38E-07
lp_1222	cps3F	polysaccharide polymerase	0.140322	4.75E-12
lp_1224	cps3G	polysaccharide biosynthesis protein (putative)	0.128029	1.19E-10
lp_1225	cps3H	polysaccharide biosynthesis protein (putative)	0.126239	1.91E-11
lp_1226	cps3I	O-acetyltransferase	0.27574	3.74E-06
lp_1227	cps3J	glycosyltransferase	0.108591	5.80E-12
lp_1231	lp_1231	oligosaccharide transporter (flippase)	0.266109	9.86E-11

lp_2099	cps4J	repeat unit transporter (flippase)	0.139545	3.25E-12
lp_2100	cps4I	glycosyltransferase	0.300885	1.06E-08
lp_2101	cps4H	polysaccharide polymerase	0.278324	2.21E-08
lp_2102	cps4G	glycosyltransferase	0.194461	1.95E-10
lp_2103	cps4F	glycosyltransferase	0.29232	1.26E-07
lp_2104	cps4E	priming glycosyltransferase	0.298552	1.45E-08
lp_2106	cps4C	polysaccharide biosynthesis protein; phosphatase (putative)	0.311304	6.36E-08
lp_2498	tagE4	poly(glycerol-phosphate) alpha-glucosyltransferase	0.338501	9.24E-06
lp_2993	lp_2993	nucleotide-binding protein, universal stress protein UspA family	0.192638	1.14E-10
lp_1848	gidC	glucose inhibited division protein GidC	0.327324	2.20E-13
lp_0735	comFA	late competence protein, ATP-dependent DNA helicase/translocase (putative)	0.333645	0.049946
lp_2244	comGE	ComG operon protein 5 precursor	0.467202	0.025277
lp_2247	comGC	ComG operon protein 3 precursor	0.456154	0.003675
lp_0403	plnR	plantaricin biosynthesis protein PlnR	0.353408	8.23E-05
lp_0404	plnL	immunity protein PlnL	0.417515	1.09E-05
lp_0405	plnK	bacteriocin precursor peptide PlnK (putative)	0.195548	1.33E-07
lp_0263	treA	trehalose-6-phosphate hydrolase	0.468996	0.000154
lp_0393	thgA1	galactoside O-acetyltransferase	0.457	0.002996
lp_2610	dxs	1-deoxy-D-xylulose-5-phosphate synthase	0.219313	2.17E-13
lp_3020	tag2	DNA-3-methyladenine glycosylase I	0.465127	0.032715
lp_0939	hsdM	type I restriction-modification system, methylation subunit	0.496332	6.44E-07
lp_2629	pox3	pyruvate oxidase	0.451145	3.52E-07
lp_3449	nox5	NADH oxidase	0.241419	2.37E-11
lp_3589	pox5	pyruvate oxidase	0.257204	2.65E-12
lp_1245	hicD2	L-2-hydroxyisocaproate dehydrogenase	0.211587	8.93E-07
lp_1280	araT1	aromatic amino acid specific aminotransferase	0.476609	1.59E-06
lp_2349	hicD3	L-2-hydroxyisocaproate dehydrogenase	0.454417	1.53E-09
lp_2738	lp_2738	L-asparaginase	0.495814	5.72E-09
lp_1005	als	acetolactate synthase	0.412726	3.80E-12
lp_1101	ldhL2	L-lactate dehydrogenase	0.345749	2.32E-06
lp_1521	lp_1521	alcohol dehydrogenase, zinc-binding (putative)	0.127516	1.91E-10
lp_3314	pflA	formate C-acetyltransferase activating enzyme	0.482609	0.000449
lp_1912	pps	phosphoenolpyruvate synthase (pyruvate phosphate dikinase)	0.449554	0.002704
lp_0070	lp_0070	4-hydroxyphenylacetate-3-hydroxylase, C-terminus	0.442017	0.000162
lp_3666	lp_3666	aromatic compound hydratase/decarboxylase	0.498926	0.026295
lp_3509	lp_3509	N-acetylglucosamine kinase (putative)	0.491029	0.002399
lp_1112	fum	fumarate hydratase	0.163421	1.33E-09
lp_1670	fabZ1	(3R)-hydroxyacyl-[acyl carrier protein] dehydratase	0.304427	1.92E-11
lp_1671	fabH2	3-oxoacyl-[acyl-carrier protein] synthase III	0.277857	1.04E-12

lp_1672	acpA2	acyl carrier protein	0.283948	6.47E-13
lp_1673	fabD	[acyl-carrier protein] S-malonyltransferase	0.256112	2.46E-14
lp_1674	fabG1	3-oxoacyl-[acyl-carrier protein] reductase	0.249288	1.27E-13
lp_1675	fabF	3-oxoacyl-[acyl-carrier protein] synthase II	0.180571	7.18E-13
lp_1676	accB2	acetyl-CoA carboxylase, biotin carboxyl carrier protein	0.171346	4.24E-13
lp_1677	fabZ2	(3R)-hydroxymyristoyl-[acyl carrier protein] dehydratase	0.196145	1.76E-11
lp_1678	accC2	acetyl-CoA carboxylase, biotin carboxylase subunit	0.207829	1.18E-13
lp_1679	accD2	acetyl-CoA carboxylase, carboxyl transferase subunit beta	0.282704	8.69E-11
lp_1680	accA2	acetyl-CoA carboxylase, carboxyl transferase subunit alpha	0.198785	8.40E-11
lp_1681	fabI	enoyl-[acyl-carrier protein] reductase (NADH)	0.300582	5.56E-11
lp_1682	lp_1682	phosphopantetheinyltransferase	0.287831	2.62E-08
lp_0371	glpD	glycerol-3-phosphate dehydrogenase, FAD-dependent	0.405765	0.01774
lp_0357	lp_0357	integral membrane protein	0.486588	3.86E-07
lp_0778	lp_0778	integral membrane protein	0.444258	2.61E-07
lp_1564	lp_1564	integral membrane protein	0.19171	1.68E-11
lp_2755	lp_2755	integral membrane protein (putative)	0.467027	6.85E-08
lp_3177	lp_3177	integral membrane protein	0.243682	0.001345
lp_3359	lp_3359	integral membrane protein	0.377134	0.000245
lp_3360	lp_3360	integral membrane protein	0.341094	5.20E-05
lp_0045	lp_0045	unknown	0.436178	2.25E-07
lp_0239	lp_0239	unknown	0.295637	1.71E-07
lp_0332	lp_0332	extracellular protein (putative)	0.430612	2.21E-10
lp_0333	lp_0333	unknown	0.497253	8.76E-09
lp_0535	lp_0535	unknown	0.327609	1.86E-07
lp_1239	lp_1239	unknown	0.478263	2.69E-08
lp_1642	lp_1642	unknown	0.465828	1.19E-06
lp_1953	lp_1953	unknown	0.161913	0
lp_2638	lp_2638	unknown	0.223134	4.78E-10
lp_3169	lp_3169	unknown	0.485828	6.59E-08
lp_0111	lp_0111	oxidoreductase	0.486124	3.79E-05
lp_0272	lp_0272	aromatic acid carboxylyase, subunit D (putative)	0.472536	1.28E-06
lp_0397	lp_0397	hydrolase, HAD superfamily, Cof family	0.479508	0.006179
lp_1947	lp_1947	acetyltransferase, GNAT family (putative)	0.388695	8.17E-08
lp_2601	lp_2601	hydrolase, HAD superfamily, Cof family	0.271126	3.19E-09
lp_3318	lp_3318	aldo/keto reductase family protein	0.470313	9.30E-06
lp_3572	lp_3572	FAD-dependent pyridine nucleotide-disulphide oxidoreductase	0.481008	3.40E-09
lp_3669	lp_3669	DegV family protein	0.423845	3.89E-09
lp_3139	lp_3139	integral membrane protein (putative)	0.207592	7.45E-06
lp_0164	lp_0164	integral membrane protein	0.143256	4.75E-14
lp_1709	lp_1709	integral membrane protein	0.450604	4.09E-06
lp_2635	lp_2635	integral membrane protein	0.341213	2.86E-07
lp_2885	lp_2885	integral membrane protein	0.124353	3.43E-08
lp_2949	lp_2949	integral membrane protein	0.334443	0.002445

lp_3180	lp_3180	integral membrane protein	0.314001	3.20E-11
lp_0032	lp_0032	unknown	0.482849	1.11E-09
lp_1166	lp_1166	unknown	0.456214	1.15E-10
lp_1168	lp_1168	unknown	0.483034	0.001428
lp_2624	lp_2624	unknown	0.469993	0.000664
lp_3668	lp_3668	acetyltransferase, GNAT family (putative)	0.494481	4.13E-06
lp_0860	lp_0860	transposase, fragment	0.449408	3.96E-05
lp_2074	lp_2074	transposase, fragment	0.40201	0.010168
lp_1574	pepE	dipeptidase	0.352504	7.44E-11
lp_1595	pepP	Xaa-Pro aminopeptidase (putative)	0.485968	1.06E-08
lp_2919	pepR2	prolyl aminopeptidase	0.338995	0.000125
lp_1063	rplQ	ribosomal protein L17	0.494195	9.09E-09
lp_1012	serS2	serine-tRNA ligase	0.483981	4.39E-09
lp_2653	rluA	ribosomal large subunit pseudouridylate synthase	0.272929	2.40E-12
lp_3319	lp_3319	nucleotide-binding protein, histidine triad family	0.496695	0.000733
lp_2726	purS	phosphoribosylformylglycinamidine synthase, PurS component	0.441363	0.001546
lp_3269	purB	adenylosuccinate lyase	0.306699	1.12E-11
lp_3270	purA	adenylosuccinate synthase	0.199682	5.73E-14
lp_3271	guaC	GMP reductase	0.169411	2.38E-13
lp_2703	pyrB	aspartate carbamoyltransferase	0.453001	0.000261
lp_2591	lp_2591	purine nucleosidase	0.26048	0
lp_0709	galE1	UDP-glucose 4-epimerase	0.365023	1.04E-08
lp_1219	glf2	UDP-galactopyranose mutase	0.201164	1.30E-14
lp_2105	cps4D	UDP N-acetyl glucosamine 4-epimerase, NAD dependent	0.351305	1.52E-08
lp_0885	bglG1	transcription antiterminator, BlgB family	0.408397	0.004692
lp_1442	lp_1442	transcription regulator, Crp/FNR family	0.473189	1.85E-05
lp_2602	ccpB	catabolite control protein B; transcriptional regulator, LacI family	0.497767	9.10E-06
lp_3470	lacR	transcription regulator. LacI family, lactose related	0.463024	4.98E-05
lp_3495	lp_3495	transcription regulator, LysR family	0.477954	5.65E-05
lp_3502	lp_3502	transcription regulator, LysR family	0.466068	0.0003
lp_1565	lp_1565	transcriptional regulator	0.262536	2.58E-09
lp_1974	lp_1974	transcription regulator of gluconeogenic genes	0.473345	4.37E-07
lp_2654	lp_2654	transcription regulator	0.355484	1.59E-08
lp_2704	pyrR1	pyrimidine operon regulator	0.391752	6.54E-12
lp_2708	pucR	purine transport regulator	0.454401	0.021238
lp_1153	lp_1153	transcription regulator, TetR family (putative)	0.486735	3.89E-08
lp_2593	rnh	ribonuclease H (putative)	0.479862	8.64E-09
lp_0424	plnH	bacteriocin ABC transporter, accessory factor PlnH	0.480418	0.000321
lp_0802	lp_0802	polar amino acid ABC transporter, substrate binding and permease protein	0.191029	0
lp_0803	glnQ1	glutamine ABC transporter, ATP-binding protein	0.217065	0

lp_1297	lp_1297	S-methylmethionine transport protein (putative)	0.472671	0.032058
lp_1745	lp_1745	D-Methionine ABC transporter, permease protein (putative)	0.34017	0.025732
lp_2312	glnH2	ABC transporter, substrate binding protein, histidine	0.309242	1.18E-11
lp_2313	glnQ4	glutamine ABC transporter, ATP-binding protein	0.357713	1.71E-08
lp_2314	glnP2	glutamine ABC transporter, permease protein	0.346077	3.78E-08
lp_3049	lp_3049	amino acid transport protein	0.412749	3.68E-09
lp_1175	glpF4	glycerol uptake facilitator protein	0.263849	1.38E-14
lp_0056	lp_0056	cation transport protein	0.499206	0.015683
lp_0400	napA1	Na(+)/H(+) antiporter	0.328074	9.24E-05
lp_1095	mtsC	manganese ABC transporter, ATP-binding protein	0.360064	2.61E-06
lp_1096	mtsB	manganese ABC transporter, permease protein	0.201112	1.30E-07
lp_1097	mtsA	manganese/zinc ABC transporter, substrate binding protein	0.335876	6.71E-08
lp_1102	citP	citrate transport protein (putative)	0.4341	5.78E-05
lp_1295	lp_1295	cation transport protein	0.30531	1.04E-06
lp_2992	mntH2	manganese transport protein	0.055519	1.09E-13
lp_3105	fhuB	iron chelatin ABC transporter, permease protein	0.436459	0.013088
lp_3106	fhuG	iron chelatin ABC transporter, permease protein	0.434239	0.001152
lp_3327	lp_3327	cadmium-/zinc-/cobalt- transporting ATPase	0.436967	8.02E-07
lp_3303	lp_3303	multidrug transport protein	0.224857	1.35E-14
lp_0116	thiP	hydroxymethylpyrimidine permease	0.48466	0.000227
lp_2710	lp_2710	purine transport protein	0.481866	3.67E-07
lp_2712	lp_2712	purine transport protein (putative)	0.366203	1.22E-12
lp_3204	nupC	pyrimidine nucleoside transport protein	0.457373	2.01E-10
lp_0259	pnuC	nicotinamide mononucleotide transporter	0.365931	1.33E-10
lp_0264	pts4ABC	PTS system, trehalose-specific IIBC component	0.390103	2.37E-07
lp_0286	pts6C	cellobiose PTS, EIIC	0.190561	8.89E-10
lp_0575	pts9AB	mannose PTS, EIIAB	0.267459	1.46E-10
lp_0576	pts9C	mannose PTS, EIIC	0.221931	2.62E-11
lp_0577	pts9D	mannose PTS, EIID	0.175639	7.76E-13
lp_3008	pts23A	cellobiose PTS, EIIA	0.44086	0.010253
lp_0217	lp_0217	ABC transporter, permease protein	0.479929	6.65E-06
lp_0455	lp_0455	transport protein	0.428832	0.000521
lp_0848	lp_0848	purine transport protein	0.381576	3.80E-07
lp_1469	sufD	ABC transporter, iron-sulfur cluster assembly protein SufD (putative)	0.459072	1.48E-05
lp_2541	lp_2541	ABC transporter, substrate binding protein	0.399199	7.29E-09
lp_2542	lp_2542	ABC transporter, permease protein (putative)	0.399594	7.96E-07
lp_2543	lp_2543	ABC transporter, ATP-binding protein	0.337626	2.60E-10
lp_2858	lp_2858	ABC transporter, ATP-binding protein, N-terminal domain	0.302136	9.77E-05
lp_1657	trpA	tryptophan synthase, beta chain	5.075436	0.004086
lp_1658	trpB	tryptophan synthase, alpha chain	3.807533	0.00020

				2
lp_1856	lp_1856	methonine synthase (cobalamine-independent), C-terminal domain (putative)	2.845339	1.12E-08
lp_0256	cbs	cystathionine beta-synthase	3.196279	2.77E-13
lp_0854	birA2	biotin--[acetyl-CoA-carboxylase] ligase and biotin operon repressor	2.271046	3.60E-13
lp_1822	gshR3	glutathione reductase	5.527294	6.66E-12
lp_2771	natC2	nicotinate phosphoribosyltransferase	2.684816	6.71E-10
lp_2578	lp_2578	adherence protein, collagen-binding domain	2.733536	6.46E-08
lp_2796	lp_2796	cell surface protein precursor	3.383017	0.000166
lp_3001	lp_3001	cell surface protein precursor (putative)	2.699509	0.008165
lp_3127	lp_3127	mucus-binding protein (putative)	2.382459	0.028629
lp_3454	lp_3454	cell surface protein (putative	4.322336	5.85E-08
lp_2396	lp_2396	extracellular protein, DUF 1093 family, membrane-bound (putative)	3.859652	0
lp_2586	lp_2586	cell surface hydrolase, DUF915 family, membrane-bound (putative)	2.127492	0.01234
lp_3025	lp_3025	extracellular protein, membrane-anchored (putative)	8.714448	9.74E-06
lp_3050	lp_3050	extracellular transglycosylase, membrane-bound (putative)	8.618547	2.69E-07
lp_3084	lp_3084	cell surface protein, ErfK family	3.261692	1.32E-05
lp_3134	lp_3134	extracellular protein, DUF 1093 family, membrane-bound (putative)	2.178896	2.59E-12
lp_0141	lp_0141	extracellular protein	2.352397	0.00688
lp_2174	lp_2174	cell surface protein precursor, DUF916 family	2.163365	0.00367
lp_2175	lp_2175	extracellular protein	2.711429	0.000385
lp_3077	lp_3077	extracellular protein (putative)	2.881764	0
lp_3450	lp_3450	extracellular protein	5.524257	1.74E-08
lp_3451	lp_3451	cell surface protein precursor	5.691818	2.18E-06
lp_3452	lp_3452	extracellular protein	5.192003	5.62E-08
lp_3453	lp_3453	extracellular protein	4.109589	3.18E-08
lp_0302	lp_0302	extracellular transglycosylase (putative)	2.04307	7.21E-11
lp_2845	lp_2845	extracellular transglycosylase (putative)	4.895575	0
lp_3014	lp_3014	extracellular transglycosylase (putative)	4.730014	0
lp_0182	lp_0182	endo-beta-N-acetylglucosaminidase	2.884872	3.62E-07
lp_1242	lp_1242	extracellular protein, NlpC/P60 family, gamma-D-glutamate-meso-diaminopimelate muropeptidase (putative)	3.693265	0.006032
lp_2520	lp_2520	extracellular protein, NlpC/P60 family, gamma-D-glutamate-meso-diaminopimelate muropeptidase (putative)	2.251393	8.99E-09
lp_3093	lp_3093	muramidase (putative)	11.65921	0
lp_1197	cps2A	polysaccharide biosynthesis protein, chain length regulator (putative)	3.108667	0.012917
lp_1198	cps2B	polysaccharide biosynthesis protein; regulator	4.185685	0.001502
lp_1199	cps2C	polysaccharide biosynthesis protein; phosphatase (putative)	3.024179	0.001604
lp_1524	ica1	glycosyltransferase	6.982087	1.19E-07
lp_1763	lp_1763	glycosyltransferase	2.088725	0.00509

lp_1816	lp_1816	D-ribitol-5-phosphate cytidylyltransferase (putative)	7.988706	1.40E-06
lp_1817	lp_1817	ribitol-5-phosphate 2-dehydrogenase (putative)	3.865604	8.33E-06
lp_2843	tagE5	poly(glycerol-phosphate) alpha-glucosyltransferase	2.990274	0
lp_2844	tagE6	poly(glycerol-phosphate) alpha-glucosyltransferase	2.559999	1.91E-13
lp_2989	gtcA3	teichoic acid glycosylation protein (putative)	2.008827	0.001889
lp_0430	txe	toxin, plasmid maintenance system killer protein	3.682038	0
lp_0431	axe	antitoxin, plasmid stabilization system protein	3.225425	3.46E-12
lp_1701	lp_1701	nucleotide-binding protein, universal stress protein UspA family	2.236655	7.91E-11
lp_1269	clpE	ATP-dependent Clp protease, ATP-binding subunit ClpE	2.22599	0.000339
lp_0129	hsp1	small heat shock protein	8.850889	0
lp_3352	hsp3	small heat shock protein	2.300624	8.40E-10
lp_0930	asp2	alkaline shock protein	2.239515	4.86E-06
lp_3128	lp_3128	stress induced DNA binding protein	2.681165	1.58E-11
lp_2078	rodA1	rod-shape determining protein	2.017418	0.000245
lp_2226	lp_2226	competence protein/transcription factor, CoiA-like family	2.647718	7.85E-10
lp_1767	lp_1767	glycosyl hydrolase, family 25 (putative)	3.269206	2.00E-06
lp_0410	plnN	bacteriocin precursor peptide PlnN (putative)	4.993239	2.08E-06
lp_0411	plnO	plantaricin biosynthesis protein PlnO	2.720956	0.000458
lp_0412	plnP	immunity protein PlnP, membrane-bound protease CAAX family	2.973296	4.26E-05
lp_3568	nanA	N-acetylneuraminate lyase	2.087221	1.51E-07
lp_3571	nanE	N-acetylmannosamine-6-phosphate 2-epimerase	2.058469	0.002494
lp_0025	malZ	maltodextrin glucosidase	2.315209	6.87E-05
lp_0027	pgmB1	beta-phosphoglucomutase	2.584304	5.62E-08
lp_0066	pgmB2	beta-phosphoglucomutase	2.959362	0.018054
lp_0193	lp_0193	exo alfa-1,4-glucosidase or trehalase	2.057565	0.01425
lp_1730	mapA	maltose phosphorylase	4.830891	9.29E-06
lp_3220	malA2	maltase-sucrase, probably sucrose-6-P hydrolase	2.999305	1.81E-06
lp_1933	thgA2	galactoside O-acetyltransferase	2.538848	0.000723
lp_0711	phnX	phosphonoacetaldehyde hydrolase	3.357587	0.007662
lp_1378	cysD	sulfate adenylyltransferase	2.187622	0.012494
lp_1379	cysC	adenylylsulfate kinase	2.080811	0.000718
lp_2109	uvrC	excinuclease ABC, subunit C	2.305482	1.27E-12
lp_3241	nth2	endonuclease III	5.389734	1.50E-09
lp_0811	lp_0811	DNA-directed DNA polymerase III, epsilon chain (putative)	2.644164	9.73E-11
lp_1858	mrr	mrr restriction system protein	2.142002	2.85E-05
lp_0159	lp_0159	short-chain dehydrogenase/oxidoreductase	2.078695	1.14E-08
lp_1975	lp_1975	short-chain dehydrogenase/oxidoreductase	2.639914	0.000671
lp_2851	lp_2851	short-chain dehydrogenase/oxidoreductase	8.129317	1.73E-06
lp_3026	lp_3026	short-chain dehydrogenase/oxidoreductase	2.077883	0.02408

lp_3029	lp_3029	oxidoreductase	3.687558	0.017052
lp_3034	lp_3034	oxidoreductase	2.027838	1.00E-06
lp_3045	lp_3045	short-chain dehydrogenase/oxidoreductase	2.53382	4.82E-13
lp_3110	lp_3110	short-chain dehydrogenase/oxidoreductase	2.549879	0.001233
lp_1721	gabT	4-aminobutyrate aminotransferase	2.078713	0.01219
lp_2683	lp_2683	aminotransferase with N-terminal regulator domain	2.889658	1.46E-12
lp_2776	dsdA	D-serine ammonia-lyase (putative)	3.28507	0
lp_2852	cmd	4-carboxymuconolactone decarboxylase (putative)	2.799283	0.000238
lp_2590	lp_2590	nitroreductase family protein	2.751678	3.01E-12
lp_1126	cydB	cytochrome D ubiquinol oxidase, subunit II	2.167444	6.17E-09
lp_3490	lp_3490	FMN-binding protein	3.070508	1.62E-13
lp_1665	adh1	alcohol dehydrogenase, zinc-binding	3.466125	5.43E-06
lp_2596	pflE	formate acetyltransferase activating enzyme (similar to)	3.286108	5.24E-13
lp_2598	pflF	formate C-acetyltransferase (similar to)	4.995373	1.12E-09
lp_3665	padA	phenolic acid decarboxylase	3.624252	1.27E-14
lp_0184	sacK1	fructokinase	41.55208	0
lp_0187	scrB	sucrose-6-P hydrolase	4.041033	7.19E-06
lp_0253	kdgK	2-keto-3-deoxygluconate kinase	2.002306	0.049891
lp_2965	lp_2965	transcriptional regulator/ sugar kinase, ROK family	2.730441	4.68E-06
lp_3011	pbg6	6-phospho-beta-glucosidase	18.76011	2.42E-10
lp_3603	lp_3603	sugar-phosphate aldolase	2.16264	0.004513
lp_3608	lp_3608	myo-inositol 2-dehydrogenase -like (promiscuous)	2.91731	0.013618
lp_3629	bgl	beta-glucosidase	2.130339	0.001123
lp_3630	lp_3630	transcriptional regulator/ sugar kinase, ROK family	2.325369	0.000865
lp_3631	lp_3631	alpha-mannosidase (promiscuous)	3.104533	1.11E-06
lp_3536	bsh1	choloylglycine hydrolase	5.518764	0
lp_1696	cfa1	cyclopropane-fatty-acyl-phospholipid synthase	3.597232	0
lp_3342	pgpA	phosphatidylglycerophosphatase (putative)	2.169706	3.89E-08
lp_0183	lp_0183	integral membrane protein	2.32459	1.58E-10
lp_0817	lp_0817	integral membrane protein	2.896361	2.89E-12
lp_0926	lp_0926	integral membrane protein	2.037964	3.66E-07
lp_0927	lp_0927	integral membrane protein	2.019926	6.22E-06
lp_1435	lp_1435	integral membrane protein	2.446112	4.85E-08
lp_1525	lp_1525	integral membrane protein	4.890936	1.43E-08
lp_1692	lp_1692	integral membrane protein	3.470083	2.65E-08
lp_1695	lp_1695	integral membrane protein	2.481481	2.82E-06
lp_1807	lp_1807	integral membrane protein	2.085335	1.26E-07
lp_1908	lp_1908	integral membrane protein	2.695066	2.19E-13
lp_2841	lp_2841	integral membrane protein	4.715405	3.71E-11
lp_3080	lp_3080	integral membrane protein	3.240793	1.81E-11
lp_3286	lp_3286	integral membrane protein	11.97454	0
lp_0224	lp_0224	integral membrane protein	5.341931	0

lp_0250	lp_0250	unknown	3.124184	0.002856
lp_0359	lp_0359	unknown	2.454955	1.08E-09
lp_0762	lp_0762	unknown	2.108845	1.96E-07
lp_0763	lp_0763	unknown	2.367383	8.20E-09
lp_0827	lp_0827	unknown	2.059878	1.08E-09
lp_0865	lp_0865	unknown	2.743501	9.75E-13
lp_0875	lp_0875	unknown	12.91159	0
lp_0928	lp_0928	unknown	2.154436	5.08E-06
lp_0934	lp_0934	PTS system IIA component	2.276087	1.13E-05
lp_1256	lp_1256	extracellular protein	3.481032	4.63E-08
lp_1347	lp_1347	unknown	2.118524	0.009947
lp_1377	lp_1377	unknown	5.715599	0.000514
lp_1708	lp_1708	unknown	2.433528	5.37E-10
lp_1762	lp_1762	unknown	4.14233	0.000159
lp_1788	lp_1788	unknown	4.009328	0
lp_1789	lp_1789	unknown	6.63385	0
lp_1913	lp_1913	unknown	2.942415	1.60E-07
lp_1929	lp_1929	unknown	2.38295	3.74E-10
lp_2085	lp_2085	unknown	2.990619	0
lp_2112	lp_2112	unknown	2.310134	2.06E-12
lp_2160	lp_2160	unknown	2.085192	1.33E-07
lp_2589	lp_2589	unknown	2.419878	2.27E-10
lp_2666	lp_2666	unknown	4.272833	1.36E-13
lp_2853	lp_2853	unknown	4.401316	2.59E-05
lp_3030	lp_3030	unknown	2.104807	0.010336
lp_3031	lp_3031	unknown	2.863469	0.027435
lp_3141	lp_3141	unknown	2.02572	1.72E-05
lp_3142	lp_3142	unknown	2.675214	1.29E-07
lp_3268	lp_3268	unknown	2.211976	0.000104
lp_3343	lp_3343	unknown	3.026198	9.00E-10
lp_3356	lp_3356	acetyltransferase, GNAT family	6.07131	0
lp_0026	lp_0026	hydrolase, HAD superfamily, Cof family	2.870945	1.74E-13
lp_0127	lp_0127	oxidoreductase	5.513273	0
lp_0146	lp_0146	NADH:flavin oxidoreductase/NADH oxidase	3.282818	1.89E-13
lp_0202	lp_0202	acetyltransferase, GNAT family (putative)	2.432928	0.003108
lp_0249	lp_0249	PTS-associated protein	3.741628	0.022154
lp_0251	lp_0251	SelA-related pyridoxal phosphate-dependent enzyme	2.895068	0.012697
lp_0311	lp_0311	acetyltransferase (putative)	3.106706	5.72E-13
lp_1380	lp_1380	phosphoesterase, DHH family	2.651285	0.000127
lp_1519	lp_1519	ATPase, AAA family (putative)	2.159664	1.41E-05

lp_1932	lp_1932	phosphohydrolase, possibly inorganic pyrophosphatase	2.192961	0.002695
lp_2003	lp_2003	zinc metallopeptidase (putative)	2.065357	1.17E-09
lp_2792	lp_2792	oxidoreductase	2.509709	3.70E-07
lp_2835	lp_2835	phosphohydrolase, HAD superfamily	2.293165	0.000675
lp_2929	lp_2929	diguanylate cyclase/phosphodiesterase, GGDEF domain (putative)	2.289796	1.32E-05
lp_2930	lp_2930	diguanylate cyclase/phosphodiesterase, EAL domain (putative)	2.437909	1.15E-10
lp_3098	lp_3098	NAD-dependent epimerase/dehydratase protein family (putative)	6.453744	1.58E-08
lp_3524	lp_3524	PTS-associated protein	2.27158	0.015669
lp_3684	lp_3684	amidohydrolase family protein	3.799276	0.003281
lp_2173	lp_2173	extracellular protein	2.144101	0.0238
lp_1552	lp_1552	integral membrane protein	2.128739	0.009829
lp_1590	lp_1590	integral membrane protein	2.954055	4.76E-08
lp_2004	lp_2004	integral membrane protein	2.043589	8.73E-12
lp_3028	lp_3028	integral membrane protein	3.107827	0.018132
lp_0207	lp_0207	unknown	2.020697	1.32E-07
lp_0327	lp_0327	unknown	2.087354	4.84E-10
lp_0554	lp_0554	unknown	2.248722	1.26E-08
lp_0828	lp_0828	unknown	2.267906	1.55E-10
lp_0835	lp_0835	unknown	2.248068	0.002408
lp_0935	lp_0935	rhamnose-like binding protein (putative)	3.145145	1.31E-09
lp_0995	lp_0995	unknown	2.46606	6.50E-13
lp_1362	lp_1362	unknown	3.828732	5.77E-12
lp_1397	lp_1397	transcription regulator (putative)	3.925949	1.59E-06
lp_1587	lp_1587	unknown	2.079208	0.006231
lp_1834	lp_1834	unknown	14.34427	6.77E-14
lp_1901	lp_1901	unknown	2.829513	1.13E-05
lp_2488a	lp_2488a	unknown	21.3516	0
lp_2880	lp_2880	unknown	2.418589	1.19E-09
lp_2883	lp_2883	unknown	2.262603	1.06E-09
lp_3032	lp_3032	unknown	3.25319	0.000582
lp_3047	lp_3047	unknown	3.310197	3.49E-12
lp_1787	cat	chloramphenicol O-acetyltransferase	3.293016	7.07E-12
lp_1687	lp_1687	GTPase	2.055799	2.85E-05
lp_1194	lp_1194	transposase, fragment (putative)	3.387858	0.002128
lp_2010	lp_2010	transposase, fragment (putative)	2.646184	0.002208
lp_2863	sip2	signal peptidase I	2.264359	0.000764
lp_0069	ptp1	protein-tyrosine phosphatase	2.257446	0.00045

lp_2840	lp_2840	ribosomal protein serine-acetylating enzyme	2.283368	0.002346
lp_2216	rpsN2	ribosomal protein S14-2	6.299764	0
lp_3120	lepA2	GTP-binding translation elongation factor LepA	7.561117	0
lp_0501	serS1	serine--tRNA ligase	4.423196	1.42E-06
lp_1289	lp_1289	purine/pyrimidine phosphoribosyltransferase (putative)	2.771468	0.008634
lp_3476	ramR	transcription regulator, AraC family, GlcNAc-like induced	3.810194	0.016377
lp_3597	rhaR	transcription regulator, AraC family, GlcNAc -like induced	2.928431	2.87E-07
lp_0133	lp_0133	transcription regulator, ArsR family	2.499392	7.87E-10
lp_2903	lp_2903	transcription regulator, ArsR family	4.816241	0
lp_0074	lp_0074	transcription regulator, DeoR family	6.148476	3.83E-12
lp_0325	lp_0325	acetoin transport repressor, GntR family	3.749453	0
lp_2651	lp_2651	transcription regulator, GntR family	5.224395	2.32E-13
lp_1857	lp_1857	transcription regulator, LysR family	3.360225	1.75E-08
lp_2689	lp_2689	transcription regulator, LysR family	3.490927	3.52E-11
lp_2804	lp_2804	transcription regulator, LysR family	5.596716	0
lp_2842	lp_2842	transcription regulator, LysR family	3.190049	1.77E-13
lp_3206	lp_3206	transcription regulator, LysR family	3.405608	0
lp_0128	lp_0128	transcription regulator, MarR family (putative)	4.256329	1.32E-14
lp_0312	lp_0312	transcription regulator, MarR family	3.180169	2.29E-13
lp_1821	lp_1821	transcription regulator, MarR family	2.133645	2.97E-09
lp_1914	lp_1914	transcription regulator, MarR family	2.95963	2.05E-13
lp_1922	lp_1922	transcription regulator, MarR family (putative)	7.484586	0
lp_1948	lp_1948	transcription regulator, MarR family	2.11697	4.25E-05
lp_3344	lp_3344	transcription regulator, MarR family	4.131906	4.67E-14
lp_0281	lp_0281	transcription regulator, MerR family	2.805684	1.63E-12
lp_2854	lp_2854	transcription regulator, MerR family	5.369504	0
lp_3013	lp_3013	transcription regulator, MerR family	12.64788	0
lp_0225	lp_0225	transcriptional regulator, Cro/CI family	6.361333	0
lp_0347	lp_0347	transcription regulator, PadR family	2.719133	2.32E-12
lp_1700	tspO	sensory protein	2.338785	1.16E-08
lp_2918	ropB	transcription regulator, TetR family	2.28982	0.000143
lp_3048	lp_3048	transcriptional regulator, Xre family (putative)	6.253254	9.53E-11
lp_3079	lp_3079	transcription regulator, PadR family (putative)	4.179842	0
lp_3172	xylR	xylose operon regulator, ROK family	3.283989	5.48E-10
lp_3415	lp_3415	transcription regulator, AraC family	2.229343	0.000468
lp_3656	srlR2	sorbitol operon transcription antiterminator, BglG family	2.013723	0.016252
lp_0162	lp_0162	transcription regulator (putative)	2.216761	2.60E-10
lp_0165	lp_0165	transcription regulator (putative)	2.020942	0.000239
lp_1688	lp_1688	transcription regulator, TetR family (putative)	2.963026	0.000947
lp_1693	lp_1693	transcription regulator, N-terminal fragment (putative)	2.877573	8.00E-07
lp_1693a	lp_1693a	transcription regulator, C-terminal fragment (putative)	2.39251	2.48E-06

lp_2772	lp_2772	transcription regulator, TetR family (putative)	3.075873	1.75E-13
lp_2902	lp_2902	transcription regulator, TetR family	9.344342	0
lp_3097	lp_3097	transcription regulator, TetR family (putative)	4.480538	9.00E-12
lp_3119	lp_3119	transcription regulator, TetR family	8.65782	0
lp_3417	lp_3417	transcription regulator, TetR family (putative)	5.03513	0
lp_3429	lp_3429	transcription regulator, TetR family (putative)	3.290224	3.80E-13
lp_0416	plnB	histidine protein kinase PlnB; sensor protein	2.326059	1.24E-07
lp_3088	hpk10	histidine protein kinase; sensor protein	2.870623	2.02E-11
lp_0223	greA1	transcription elongation factor GreA	2.198512	7.73E-10
lp_0502	sdaC	serine transporter	2.548507	3.36E-08
lp_1409	lp_1409	amino acid efflux protein	2.578959	0.000365
lp_1722	lp_1722	4-aminobutanoate transport protein	2.825301	0.002106
lp_0712	phnE1	phosphonates ABC transporter, permease protein	2.084205	0.00162
lp_0326	lp_0326	acetoin ABC transporter, ATP-binding protein	2.451889	3.23E-13
lp_0498	deoP	deoxyribose transporter	2.327734	0.000239
lp_1729	malT	GPH family transporter, carbohydrate proton symport, maltose	5.199867	2.78E-05
lp_3533	lp_3533	GPH family transporter, carbohydrate symport, maltose-isomaltose (or trehalose), not sucrose	4.084354	0.002444
lp_3604	iolT1	MFS family transporter, carbohydrate, myo-inositol and similar	2.074491	3.91E-07
lp_3398	pacL3	cation transporting P-type ATPase	4.090358	3.37E-09
lp_3435	cadA	cadmium transporting P-type ATPase	5.102403	3.96E-05
lp_1910	drrA	ABC transporter, ATP-binding protein	7.548852	3.16E-11
lp_3284	qacC	quaternary ammonium compound-resistance protein	2.788903	1.29E-12
lp_3285	qacH	quaternary ammonium compound-resistance protein	2.450829	5.03E-09
lp_0185	pts1BCA	PTS, EIIBCA, oligosucrose	6.882913	3.35E-09
lp_2647	pts19A	N-acetylglucosamine/galactosamine PTS, EIIA	3.071015	1.98E-10
lp_2648	pts19D	N-acetylgalactosamine PTS, EIID	2.802361	1.04E-09
lp_2649	pts19C	N-acetylgalactosamine PTS, EIIC	3.661948	3.28E-12
lp_2650	pts19B	N-acetylgalactosamine PTS, EIIB	3.4686	3.77E-10
lp_3009	pts23B	cellobiose PTS, EIIB	3.007369	0.006142
lp_3010	pts23C	cellobiose PTS, EIIC	9.147068	0.000213
lp_3542	pts34A	PTS, EIIA	2.248171	0.017906
lp_3543	lp_3543	bifunctional protein: transcriptional regulator; PTS, EIIA	3.003182	1.68E-10
lp_3601	pts36C	galactitol PTS, EIIC	2.634056	0.027818
lp_0134	lp_0134	transport protein	2.255047	3.28E-07
lp_0160	lp_0160	ABC transporter, ATP-binding protein	4.683747	3.25E-12
lp_0161	lp_0161	ABC transporter, permease protein	8.123782	1.11E-12
lp_0298	lp_0298	ABC transporter, permease protein	2.168139	0.038594
lp_0299	lp_0299	ABC transporter, ATP-binding protein	2.441288	0.010286
lp_0348	lp_0348	transport protein	2.832902	3.47E-11
lp_0492	lp_0492	transport protein	2.302984	7.78E-06

lp_0831	lp_0831	transport protein, C-terminal domain	2.529948	0.00111
lp_0894	lp_0894	transport protein	4.246925	2.50E-14
lp_1689	lp_1689	transport protein	3.80094	0.006276
lp_1921	lp_1921	transport protein	4.413661	2.23E-12
lp_2084	lp_2084	transport protein, MFS (putative)	8.849085	1.80E-11
lp_2394	lp_2394	ABC transporter, ATP-binding and permease protein	2.003572	1.01E-05
lp_2497	lp_2497	ABC transporter, ATP-binding and permease protein	2.274559	0.005975
lp_2688	lp_2688	transport protein	2.424328	0.000556
lp_2739	lp_2739	ABC transporter, ATP-binding protein	2.231544	7.48E-08
lp_2740	lp_2740	ABC transporter, permease protein	2.456407	9.64E-07
lp_2773	lp_2773	ABC transporter, permease protein	16.36422	0
lp_2774	lp_2774	ABC transporter, ATP-binding protein	12.08176	0
lp_2822	lp_2822	ABC transporter, permease protein	8.67301	0
lp_2823	lp_2823	ABC transporter, ATP-binding protein	11.63454	0
lp_2901	lp_2901	ABC transporter (putative)	8.820371	0
lp_3101	lp_3101	transport protein	17.32739	0

[a] Genes with more than 2-fold upregulation or downregulation were included.
[b] P-value cut-off was 0.05
