# Supplementary material for: Transcriptome signatures of class I and III stress response deregulation in Lactobacillus plantarum reveal pleiotropic adaptation
Source: Microb Cell Fact. 2013 Nov 18;12:112. doi: 10.1186/1475-2859-12-112 (PMC3842655; doi:10.1186/1475-2859-12-112)
Supplement: Additional file 3: Table S2 — Abbreviations used in Figure 4. [file 1475-2859-12-112-S3.pdf]

Supplementary information related to:

Transcriptome signatures of class I and III stress response deregulation in *Lactobacillus plantarum* reveal pleiotropic adaptation

Running title: *ctsR* and *hrcA* deregulation in *L. plantarum* WCFS1

Hermien van Bokhorst-van de Veen, Roger S. Bongers, Michiel Wels, Peter A. Bron, and Michiel Kleerebezem

Supplementary information : Abbreviations used in Fig. 4

**Abbreviation**	**Component**
ACALD	acetaldehyde dehydrogenase (acetylating)
ACALDt	acetaldehyde reversible transport
ACKr	acetate kinase
ACLDC	acetolactate decarboxylase
ACLS	acetolactate synthase (Also catalyzes ACHBS)
ACt6	acetate transport in/out via proton symport
ACTNdiff	(R)-acetoin diffusion
ACTPASE	acylphosphatase
ALCD19	alcohol dehydrogenase (glycerol)
ALCD2x	alcohol dehydrogenase (ethanol: NAD)
ALDD2x	aldehyde dehydrogenase (acetaldehyde, NAD)
ALDD8x	aldehyde dehydrogenase (D-glyceraldehyde, NAD)
ALOX	oxidative decarboxylation of acetolacate (chemical)
ATPM	ATP maintenance requirement
BTDD-RR	(R,R)-butanediol dehydrogenase
BTDt1-RR	(R,R)-butanediol transport in/out via diffusion reversible
CITL	citrate lyase
CITt6	citrate transport in/out via proton symport
CRCT	CTP:D-ribitol-5-phosphate cytidylyltransferase
DHAPT	dihydroxyacetone phosphotransferase
DHAt	dihydroxyacetone transport via facilitated diffusion
DIACTt	diacetyl diffusion
ENO	enolase

ETOHt1	ethanol transport in/out via diffusion
F6PA	fructose-6-phosphate aldolase
FBA	fructose-bisphosphate aldolase
FORt2	formate transport in via proton symport
FRDx	fumarate reductase (NADH)
FUM	fumarase
G3PD1	glycerol-3-phosphate dehydrogenase (NAD)
G3PD4	glycerol-3-phosphate dehydrogenase (NAD)
G3PO	glycerol 3-phosphate oxidase
G6PDHy	glucose 6-phosphate dehydrogenase
GAPD	glyceraldehyde-3-phosphate dehydrogenase (NAD)
GLCNt2	D-gluconate transport via proton symport
GLCpts	D-glucose transport via PEP:Pyr PTS
GLYCK	glycerate kinase
GLYCt1	glycerol transport via uniport (facilitated diffusion)
GLYK	glycerol kinase
GNK	gluconokinase
LAR	lactate racemase
LDH_D	D-lactate dehydrogenase
LDH_L	L-lactate dehydrogenase
L-LACt2	L-lactate reversible transport via proton symport
MALLAC	malolactic enzyme
MDH	malate dehydrogenase
ME1x	malic enzyme (NAD)
NADH4	NADH dehydrogenase (Menaquinone 7 & no proton)

NOX1	NADH oxidase (H2O2 forming)
NOX2	NADH oxidase (H2O forming)
NPR	NADH peroxidase
PC	pyruvate carboxylase
PDH	pyruvate dehydrogenase
PFK	phosphofructokinase
PFL	formate C-acetyltransferase
PGDH	phosphogluconate dehydrogenase
PGI	glucose-6-phosphate isomerase
PGK	phosphoglycerate kinase
PGL	6-phosphogluconolactonase
PGM	phosphoglycerate mutase
PKL	phosphoketolase
PPCK	phosphoenolpyruvate carboxykinase
PPS	phosphoenolpyruvate synthase
PRPPS	phosphoribosylpyrophosphate synthetase
PTAr	phosphotransacetylase
PYK	pyruvate kinase
PYROX	pyruvate oxidase
PYRt2	pyruvate reversible transport via proton symport
RBK	ribokinase
RBLK2	L-ribulokinase (ribitol)
RBT5PDHy	ribitol-5-phosphate 2-dehydrogenase (NADP)
RIBt2	ribose transport in via proton symporter
RPE	ribulose 5-phosphate 3-epimerase

RPI	ribose-5-phosphate isomerase
SUCCt6	succinate transporter in/out via proton symport
TAL	transaldolase
TKT1	transketolase
TKT2	transketolase
TPI	triose-phosphate isomerase
